# Hydroxychloroquine Inhibits the Trained Innate Immune Response to Interferons

**DOI:** 10.1016/j.xcrm.2020.100146

**Published:** 2020-11-10

**Authors:** Nils Rother, Cansu Yanginlar, Rik G.H. Lindeboom, Siroon Bekkering, Mandy M.T. van Leent, Baranca Buijsers, Inge Jonkman, Mark de Graaf, Marijke Baltissen, Lieke A. Lamers, Niels P. Riksen, Zahi A. Fayad, Willem J.M. Mulder, Luuk B. Hilbrands, Leo A.B. Joosten, Mihai G. Netea, Michiel Vermeulen, Johan van der Vlag, Raphaël Duivenvoorden

**Affiliations:** 1 Department of Nephrology, Radboud Institute for Molecular Life Sciences, Radboud University Medical Center, Nijmegen, the Netherlands; 2 Department of Molecular Biology, Faculty of Science, Radboud Institute for Molecular Life Sciences, Oncode Institute, Radboud University Nijmegen, Nijmegen, the Netherlands; 3 Department of Internal Medicine, Radboud Institute for Molecular Life Sciences, Radboud University Medical Center, Nijmegen, the Netherlands; 4 Biomedical Engineering and Imaging Institute, Icahn School of Medicine at Mount Sinai, New York, NY, USA; 5 Department of Medical Biochemistry, Amsterdam University Medical Centers, Amsterdam, the Netherlands; 6 Laboratory of Chemical Biology, Department of Biomedical Engineering and Institute for Complex Molecular Systems, Eindhoven University of Technology, Eindhoven, the Netherlands; 7 Department of Oncological Sciences, Icahn School of Medicine at Mount Sinai, New York, NY, USA; 8 Department of Immunology and Metabolism, Life and Medical Sciences Institute (LIMES), University of Bonn, Bonn, Germany

**Keywords:** COVID-19, SARS-CoV-2, hydroxychloroquine, trained immunity, interferon, chloroquine, innate immune memory, lipidome, monocytes

## Abstract

Hydroxychloroquine is being investigated for a potential prophylactic effect in severe acute respiratory syndrome coronavirus 2 (SARS-CoV-2) infection, but its mechanism of action is poorly understood. Circulating leukocytes from the blood of coronavirus disease 2019 (COVID-19) patients show increased responses to Toll-like receptor ligands, suggestive of trained immunity. By analyzing interferon responses of peripheral blood mononuclear cells from healthy donors conditioned with heat-killed *Candida*, trained innate immunity can be modeled *in vitro*. In this model, hydroxychloroquine inhibits the responsiveness of these innate immune cells to virus-like stimuli and interferons. This is associated with a suppression of histone 3 lysine 27 acetylation and histone 3 lysine 4 trimethylation of inflammation-related genes, changes in the cellular lipidome, and decreased expression of interferon-stimulated genes. Our findings indicate that hydroxychloroquine inhibits trained immunity *in vitro*, which may not be beneficial for the antiviral innate immune response to SARS-CoV-2 infection in patients.

## Highlights

PBMCs of COVID-19 patients show increased responses to Toll-like receptor ligands
Trained immunity is modeled *in vitro* using *Candida*-trained PBMCs
Hydroxychloroquine inhibits changes in lipidome and histone modifications
Hydroxychloroquine dampens the trained response to interferons and viral stimuli

## Graphical Abstract

**Figure:**
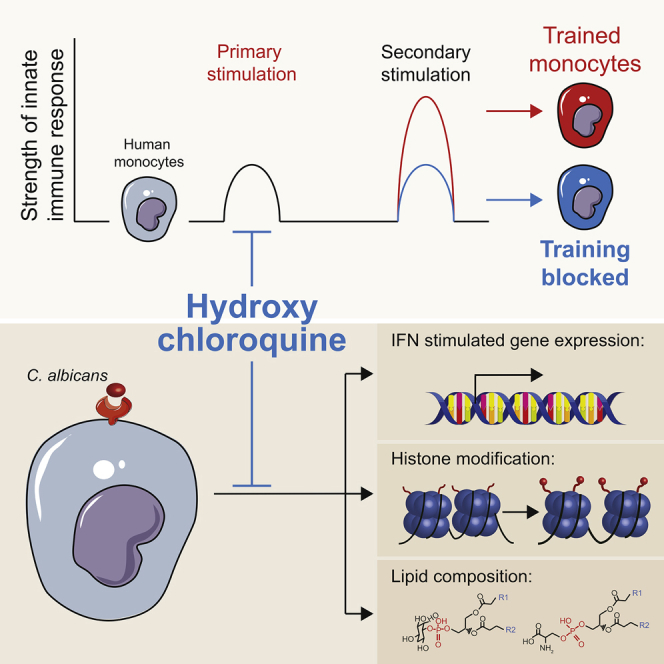

## Teaser

Peripheral blood mononuclear cells (PBMCs) of COVID-19 patients show increased responses to Toll-like receptor ligands, suggestive of innate immune reprogramming. Rother et al. show that hydroxychloroquine inhibits the interferon response of *Candida*-trained PBMCs from healthy donors *in vitro* and blocks associated changes in lipidome and histone modifications.

## Introduction

The severe acute respiratory syndrome coronavirus 2 (SARS-CoV-2), which causes coronavirus disease 2019 (COVID-19), has spread globally since the December 2019 outbreak in China. The majority of COVID-19 patients have mild symptoms, but some develop severe pneumonia.^1^ The factors that cause severe illness are not fully understood, but a growing body of evidence points to an inadequate immune response, and previous studies have shown that coronaviruses have multiple strategies to evade innate immune sensing.^2^ This is exemplified by the fact that in COVID-19 patients, a decreased type I interferon (IFN) response is observed, which is associated with impaired viral clearance.^3^^,^^4^ Ineffective clearance of SARS-CoV-2 may lead to uncontrolled tissue inflammation and poor outcome.

To date, no specific therapy is available to treat COVID-19. The antimalarial drugs chloroquine and hydroxychloroquine have been proposed as prophylactic and therapeutic agents.^5^, ^6^, ^7^ These drugs were observed to inhibit SARS-CoV-2 viral replication *in vitro* in primate cells.^8^ However, there is no confirmation so far that these drugs can affect viral replication *in vivo* in humans.^9^ Chloroquine and hydroxychloroquine also have immunomodulating properties, which may influence the disease course of COVID-19.^10^ Chloroquine and hydroxychloroquine treatment of COVID-19 is a topic of intense debate and investigation, especially in the context of prophylaxis. Their use remains controversial, as there is no clear evidence of their efficacy and a poor understanding of their mode of action.^11^^,^^12^ Better knowledge of how these 4-aminoquinolines affect the immune response is fundamentally important to uncover whether these drugs can, or cannot, be beneficial in the prevention or treatment of COVID-19.

In the current study, we investigated the immune response in COVID-19 and the immunomodulatory properties of hydroxychloroquine. Using an integrative approach with functional and transcriptomic analyses, we show marked alterations in the function and phenotype of monocytes isolated from COVID-19 patients and show IFN-stimulated genes to be associated with disease severity. By combining transcriptomic, metabolomic, and epigenetic studies, we reveal that hydroxychloroquine can prevent the induction of trained immunity. Trained immunity is a functional adaptation of monocytes induced by epigenetic reprogramming that potentiates their immunologic response.^13^ Our findings provide insight into the mechanism of action of hydroxychloroquine and indicate it decreases the trained innate immune response, including to virus-like stimuli and IFNs.

## Results

### Monocyte Phenotype and Function in COVID-19

We studied 13 patients who were admitted to Radboud University Medical Center, a tertiary care university hospital, with a SARS-CoV-2 infection. Patients were included if they were older than 18 years of age and diagnosed with COVID-19. Blood was obtained at admission and at 5 days after admission in patients who were still hospitalized. Treatment with chloroquine was started at the time of admission and continued for 5 days. The median age was 68 years (interquartile range [IQR], 54–73). Five patients had a history of pulmonary disease, three of cardiovascular disease, and three of malignancy. Most patients presented with fever (62%), cough (77%), and/or dyspnea (54%). Seven of the 13 patients required oxygen supplementation at presentation (all ≤5 L/min). All patients had signs of pneumonitis on chest imaging. None of the patients were critically ill at the time of presentation. Patient characteristics are shown in Table S1, and complete blood counts of all subjects are shown in Table S2.

**Figure 1:**
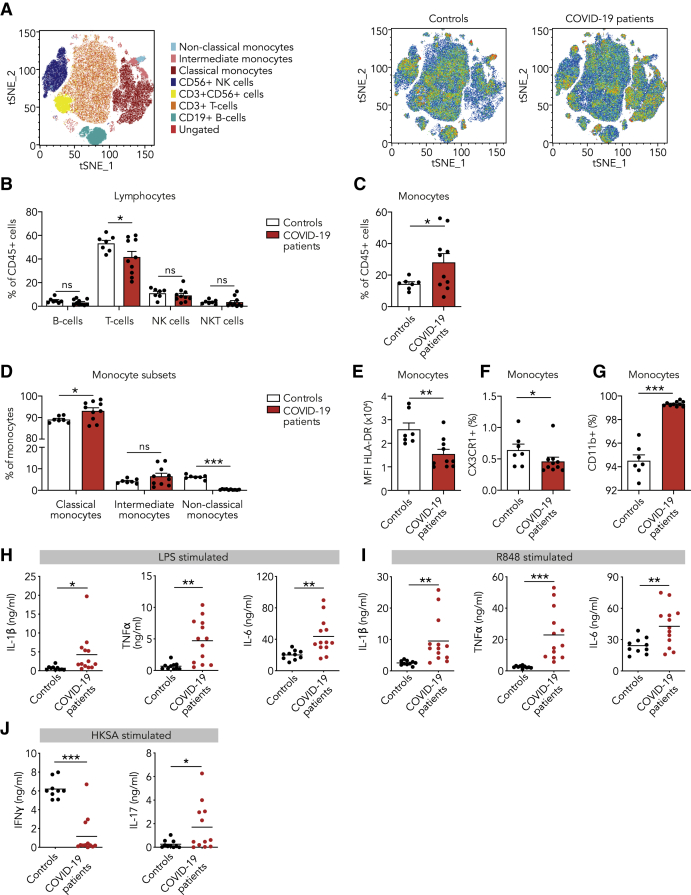
Innate Immune Response in COVID-19 Patients at the Time of Admission (A–G) PBMCs isolated from COVID-19 patients at admission and from healthy controls were analyzed using flow cytometry (n = 10 for COVID-19 patients, n = 7 for healthy controls). (A) t-distributed stochastic neighbor embedding (tSNE) plots showing unsupervised clustering on the expression of 10 markers (CD45, CD14, CD16, CD3, CD19, CD56, HLA-DR, CD11b, CCR2, and CX3CR1) in controls and COVID-19 patients. (B) Quantification of lymphocytes using gating strategy shown in Figure S1C indicated decreased amounts of T cells in COVID-19 patients. (C and D) Quantification of monocytes showed overall higher counts in COVID-19 patients that was due to higher number of classical monocytes (CD14^2+^,CD16^−^), whereas non-classical monocytes (CD14^+^, CD16^2+^) were reduced in COVID-19 patients. (E–G) Analysis of marker expression on monocytes revealed reduced expression of HLA-DR (E), reduced number of CX3CR1-expressing monocytes (F), and increased number of CD11b-expressing monocytes (G) in COVID-19 patients. (H and I) Isolated PBMCs were stimulated with LPS (H) or R848 (I) for 24 h, after which the production of IL-1β, IL-6, and TNF-α was quantified in the supernatant using ELISA. COVID-19 patient PBMCs show increased cytokine production upon stimulation with either stimulus (n = 13 for COVID-19 patients, n = 10 for healthy controls). (J) Isolated PBMCs were stimulated with heat-killed *Staphylococcus aureus* (HKSA) for 7 days, after which the production of IFNγ and IL-17 was quantified using ELISA. IFNγ response was reduced, whereas IL-17 production was elevated in COVID-19 patients. (n = 12 for COVID-19 patients, n = 10 for healthy controls) Data are presented as mean ± SEM.∗p < 0.05, ∗∗p < 0.01, ∗∗∗p < 0.001 for two-sided Student’s t test (for normally distributed data) or Kruskal-Wallis test.

We investigated the immune response in COVID-19 patients and compared it to healthy controls. For this purpose, peripheral blood mononuclear cells (PBMCs) were isolated from the blood, and immune cell subsets were analyzed by flow cytometry (Figure 1A; Figures S1B and S1C). At the time of admission, patients with COVID-19 had slightly fewer T lymphocytes, but no differences in other lymphocyte subsets (Figure 1B). Monocytes were markedly increased in COVID-19 patients, mainly due to a striking increase in CD14^2+^CD16^−^ (classical) monocytes (Figures 1C and 1D). Interestingly, CD14^+^CD16^2+^ (non-classical) monocytes were hardly detectable in COVID-19 patients (Figure 1D), which corroborates recent reports by others.^14^ Human leucocyte antigen DR (HLA-DR) was reduced on monocytes from COVID-19 patients (Figure 1E). Low HLA-DR expression was recently shown to be associated with monocyte hyperactivation and excessive release of interleukin-6 (IL-6) in COVID-19 patients.^15^ Expression of CX3CR1, which is involved in monocyte chemotactic migration and is mostly expressed by the non-classical monocyte subset,^16^ was reduced (Figure 1F), in accordance with the observed decrease in non-classical monocytes (Figure 1D). The integrin CD11b, a marker of monocyte activation, was upregulated on monocytes of COVID-19 patients (Figure 1G). Lymphocyte and monocyte subsets as well as HLA-DR, CX3CR1, and CD11b expression did not change over the course of 5 days in patients who remained hospitalized (Figures S2A–S2G).

We performed functional assays by stimulating PBMCs for 24 h *ex vivo* and subsequently measuring cytokine release, namely IL-1β, IL-6, and tumor necrosis factor alpha (TNF-α). We observed markedly elevated cytokine responses in COVID-19 patients upon Toll-like receptor 4 (TLR4) activation by lipopolysaccharide (LPS) and TLR7/8 activation by R848 (Figures 1H and 1I). Enhanced cytokine responses were also observed upon stimulation with the TLR2 agonist Pam3CSK4 and heat-killed *Candida albicans* (HKCA) (Figures S1D and S1E). This increased cytokine response was unchanged in patients who remained hospitalized throughout our 5-day observation period (Figure S2H).

Next, we explored if the changes in the innate immune profile were associated with altered responses in the adaptive immune system. For this purpose, we stimulated PBMCs for 7 days with heat-killed *Staphylococcus aureus* (HKSA) and measured IFNγ as a marker of T helper cell 1 (Th1) activation and IL-17 as a marker of Th17 activation (Figure 1J). In healthy controls, we observed substantial IFNγ and little IL-17 production, indicating a dominant Th1 response. In contrast, IFNγ production was reduced and IL-17 production enhanced in COVID-19 patients, indicating polarization toward a Th17 response.

### IFN-Stimulated Gene Expression Is Related to the Development of Severe Disease

**Figure 2:**
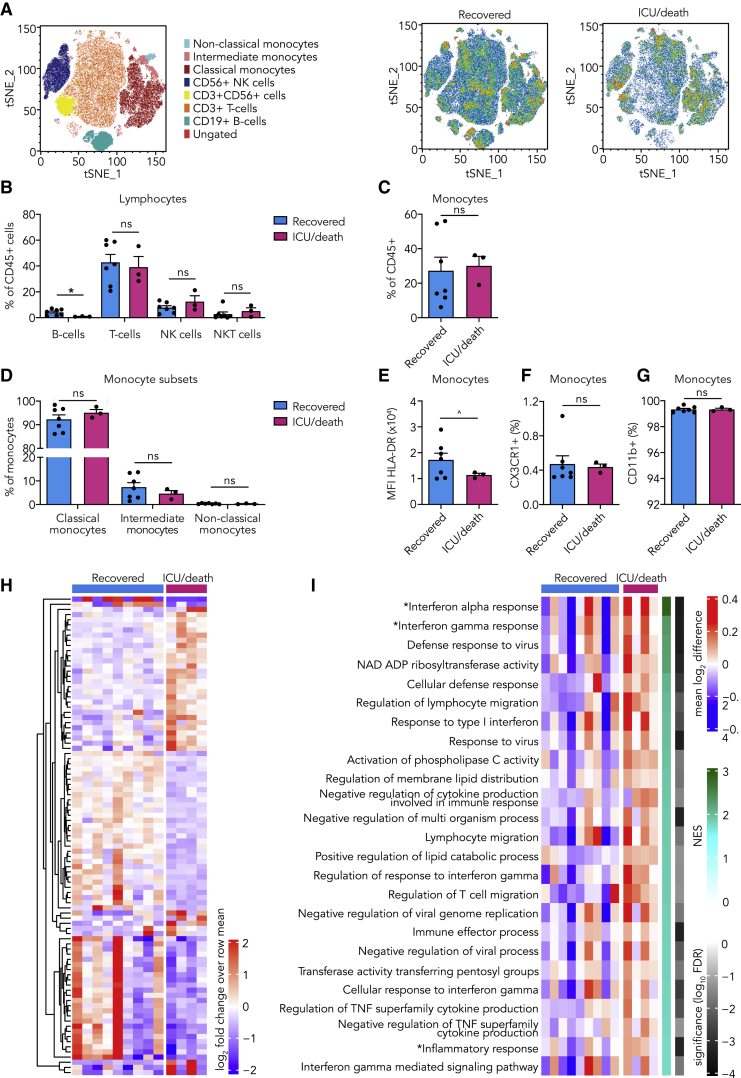
Innate Immune Response in COVID-19 Patients at Presentation and the Relation to Outcome (A–G) PBMCs isolated from COVID-19 patients at admission were analyzed using flow cytometry (n = 7 for COVID-19 patients who recovered, n = 3 for COVID-19 patients who eventually required ICU admission or died). (A) tSNE plots showing unsupervised clustering on the expression of 10 markers (CD45, CD14, CD16, CD3, CD19, CD56, HLA-DR, CD11b, CCR2, and CX3CR1) in COVID-19 patients who recovered versus those who eventually required ICU admission or died. (B) Quantification of lymphocytes using gating strategy shown in Figure S1C indicated minor differences in B cells between COVID-19 patients who recovered versus those who eventually required ICU admission or died. (C and D) Quantification of monocytes showed no difference between COVID-19 patient groups. (E–G) Expression of HLA-DR (E), CX3CR1 (F), and CD11b (G) on monocytes did not differ between COVID-19 patient groups. (H and I) Transcriptome analysis was performed on isolated monocytes of COVID-19 patients at admission. (n = 9 for COVID-19 patients who recovered, n = 4 for COVID-19 patients who required ICU admission or died) (H) Heatmap of differentially expressed genes (p < 0.005) (listed in Table S3) between COVID-19 patients who recovered versus those who eventually required ICU admission or died. (I) Heatmap of Gene Ontology (GO) pathways and hallmark pathways that are significantly enriched (false discovery rate [FDR], <0.05) in COVID-19 patients who recovered versus those who eventually required ICU admission or died (∗, hallmark pathways; NES, normalized enrichment score). Data are presented as mean ± SEM; ˆp < 0.06, ∗p < 0.05 for two-sided Student’s t test (for normally distributed data) or Kruskal-Wallis test.

Of the 13 patients with COVID-19 that were included in our study, nine recovered without requiring intensive care unit (ICU) admission, and four required ICU admission (n = 3) or died (n = 1) (Figure S1A). At the time of presentation, we observed no clear differences in clinical variables between patients who recovered and those who required ICU admission or died (Table S1). We were interested if we could detect immune response differences at admission that could be related to patient outcome. No differences in leucocyte subsets between both groups was observed, except for a lower B cell count in patients who eventually required ICU admission or died (Table S2; Figures 2A–2D). Monocyte HLA-DR expression was reduced, indicating that an inflammatory monocyte phenotype was more pronounced in patients who eventually required ICU admission or died (Figure 2E). CX3CR1 and CD11b expression were equal in both groups (Figures 2F and 2G). Next, we isolated monocytes of the COVID-19 patients and analyzed their transcriptomes by RNA sequencing. We found marked differences in transcription of IFN-stimulated genes, which are critical in the context of viral infections.^17^ Notably, higher expression of IFN-stimulated genes was associated with the eventual ICU admission or death (Figures 2H and 2I; Table S3).

**Figure 3:**
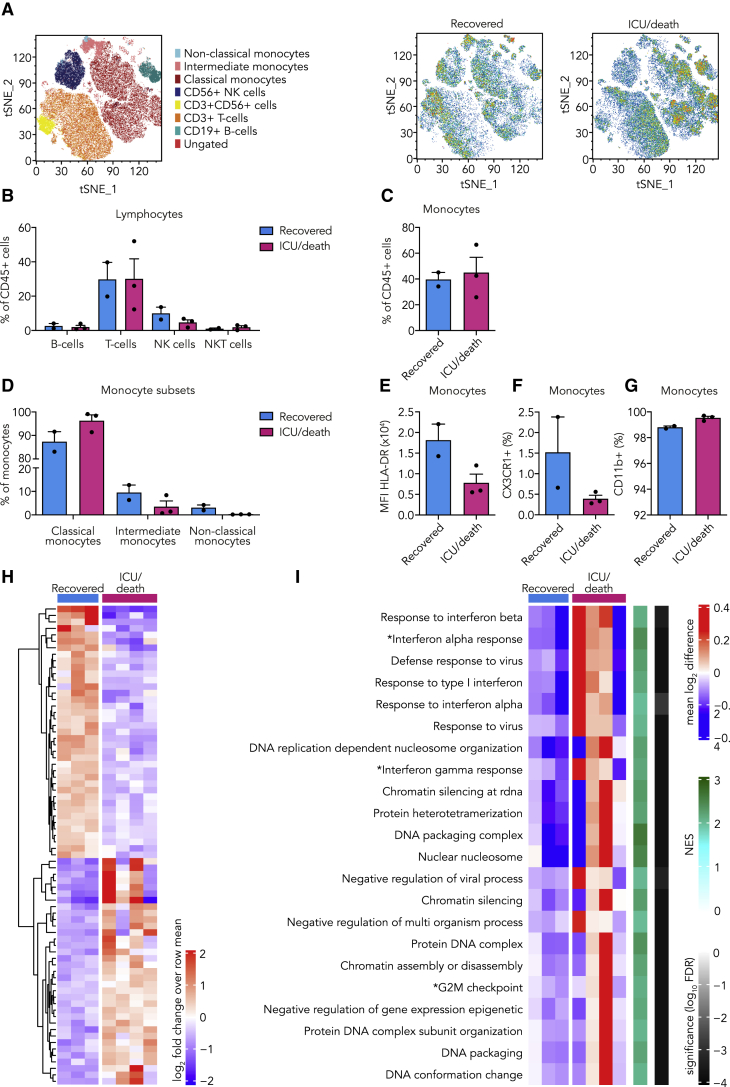
Innate Immune Response in COVID-19 Patients 5 Days after Admission and the Relation to Outcome (A–G) PBMCs isolated from COVID-19 patients 5 days after admission were analyzed using flow cytometry (n = 2 for COVID-19 patients who recovered, n = 3 for COVID-19 patients who required ICU admission or died). (A) tSNE plots showing unsupervised clustering on the expression of 10 markers (CD45, CD14, CD16, CD3, CD19, CD56, HLA-DR, CD11b, CCR2, and CX3CR1) in COVID-19 patients who recovered versus those who required ICU admission or died. (B) Quantification of lymphocytes using gating strategy shown in Figure S1C indicated no differences between both COVID-19 patient groups. (C) Quantification of overall monocytes showed no difference between COVID-19 patient groups. (D) Subset analysis of monocytes revealed increased amounts of non-classical monocytes (CD14^+^, CD16^2+^) in COVID-19 patients who recovered compared to those who required ICU admission or died. (E and F) Expression of HLA-DR (E) and numbers of CX3CR1-expressing monocytes (F) were increased in COVID-19 patients who recovered compared to those who required ICU admission or died. (G) CD11b expression on monocytes did not differ between both COVID-19 patient groups. (H and I) Transcriptome analysis was performed on isolated monocytes of COVID-19 patients 5 days after admission. (n = 3 for COVID-19 patients who recovered, n = 4 for COVID-19 patients who required ICU admission or died). (H) Heatmap of differentially expressed genes (p < 0.001) (listed in Table S4) between COVID-19 patients who recovered versus those who required ICU admission or died. (I) Heatmap of GO pathways and hallmark pathways that are significantly enriched (FDR, <0.01) in COVID-19 patients who recovered compared to those who required ICU admission or died (∗, hallmark pathways). Data are presented as mean ± SEM.

Six patients recovered fast and were discharged within the first 5 days, whereas seven patients remained hospitalized. We obtained PBMCs from this latter group 5 days after admission. At this time point, a clear distinction could be made, based on clinical parameters, between patients who recovered versus those who required ICU admission or died (Table S1). No differences in lymphocyte subsets were observed (Figures 3A and 3B; Figure S3). However, patients who eventually required ICU admission or died had more classical monocytes (Figures 3C and 3D). Importantly, the number of non-classical monocytes restored in patients who would recover but remained virtually undetectable in patients that required ICU admission or died (Figure 3D). Clear differences were visible in monocyte surface marker expression, with decreased HLA-DR and CX3CR1 expression in patients who eventually required ICU admission or died (Figures 3E and 3F). There was no difference in CD11b expression between the groups (Figure 3G). Transcriptome analysis of circulating monocytes showed a clear distinction between both patient groups. Similar to what we observed at the time of admission, we found enhanced transcription of IFN-stimulated genes 5 days after admission in patients who eventually required ICU admission or died (Figures 3H and 3I).

Taken together, these immune profiling data show that the inflammatory response in SARS-CoV-2 infection is characterized by marked alterations in the innate immune system, a result that corroborates previous reports.^15^^,^^18^, ^19^, ^20^ Monocytes show signs of enhanced activation and increased expression of IFN-stimulated genes, which are likely markers of disease severity, as we found them to be associated with a poor outcome. Importantly, we revealed an elevated monocyte-derived cytokine response to *ex vivo* stimulation of TLR2, TLR4, and TLR7/8. This enhanced responsiveness, which we observed to persist during the course of the disease, is reminiscent of the inflammatory phenotype previously reported in sepsis and influenza. The enhanced innate immune response is indicative of innate immune reprogramming, which is a mechanism that contributes to improved anti-viral mechanisms and resolution of infection.^13^^,^^21^^,^^22^

### Hydroxychloroquine Prevents the Induction of Trained Immunity

**Figure 4:**
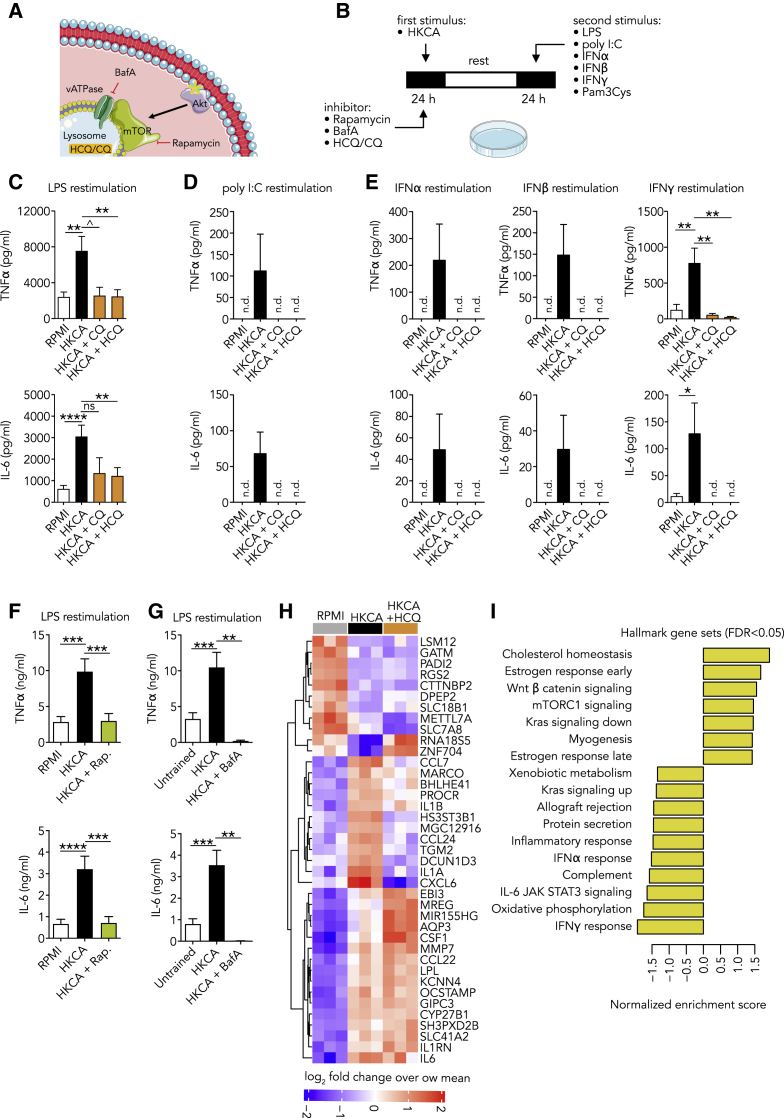
Hydroxychloroquine (HCQ) Prevents the Induction of Trained Immunity (A) Schematic representation of the interaction between the lysosome and the AKT/mTOR signaling pathway. (B) Schematic representation of the trained immunity assay. (C–E) PBMCs were stimulated for 24 h with HKCA with or without specified inhibitors or RPMI as a control. After a 5-day resting period, cells were restimulated for 24 h, and cytokine production was measured in the supernatant. (C) HCQ and chloroquine prevent the induction of a trained immune response to LPS (B, n = 7–17). (D and E) HCQ and chloroquine prevent the induction of a trained immune response to poly I:C (D, n = 5), IFNα (E, n = 5), IFNβ (E, n = 5), and IFNγ (E, n = 5-8, n.d., not detectable). (F) mTOR inhibition prevents the induction of a trained immunity response to LPS (n = 11). (G) Inhibiting lysosome acidification with bafilomycin A prevents the induction of a trained immune response to LPS (n = 4–9). (H and I) PBMCs were stimulated with HKCA, HKCA+HCQ, or RPMI as a control for 24 h. Subsequently, monocytes were purified and transcriptome analysis was performed. (n = 3 for each treatment group) (H) Heatmap of most significantly changing genes between HKCA-stimulated and control PBMCs. (I) Pathway analysis of most significant genes identified (FDR, <0.05) between HKCA-treated cells and HKCA+HCQ-treated cells. Normalized enrichment score is shown for HKCA+HCQ and HKCA, with positive values showing pathways upregulated in HKCA and negative values indicating pathways upregulated in HKCA+HCQ. Data are presented as mean ± SEM; ˆp < 0.06, ∗p < 0.05, ∗∗p < 0.01, ∗∗∗p < 0.001; one-way ANOVA with Dunnett’s post-test.

The data presented thus far revealed enhanced responsiveness of monocytes in patients with active COVID-19. Such functional adaptation of monocytes is also observed in processes like priming and trained immunity, which potentiate the anti-viral innate immune response.^13^^,^^23^ This result prompted us to investigate whether 4-aminoquinolines can affect trained immunity. Chloroquine and hydroxychloroquine are weak bases that passively diffuse to the lysosome, where they interfere with its function.^10^ Lysosomes are at the center of coordinating immunometabolism and the innate immune response by mammalian target of rapamycin (mTOR), which is activated at the lysosomal membrane (Figure 4A).^24^ Interestingly, activation of key regulators of lysosome genes is characteristic of the trained macrophage phenotype and distinguishes it from its LPS-tolerized counterpart.^25^

To investigate the effect of chloroquine (CQ) and hydroxychloroquine (HCQ) on trained immunity, we adapted a previously described *in vitro* protocol in which human PBMCs are stimulated with RPMI (control) or HKCA for 24 h.^26^ HKCA is a well-described stimulus to induce trained immunity but can also be induced by other stimuli, such as IL-1β. The cells were subsequently washed and rested for 5 days in culture medium, followed by a second 24-h stimulus (LPS, Pam3CSK4 poly I:C, IFNα, IFNβ, or IFNγ) (Figure 4B). We observed that HKCA-trained cells produced markedly more cytokines upon restimulation with either LPS or Pam3CSK4. This effect was abrogated when cells were treated with chloroquine and hydroxychloroquine for 24 h during HKCA stimulation, indicating that these compounds prevent the induction of trained immunity (Figure 4C; Figure S4A). To exclude that chloroquine and hydroxychloroquine inhibit cytokine production in general, we treated untrained monocytes with chloroquine and hydroxychloroquine for 24 h. Cytokine production upon LPS stimulation 5 days later was not affected (Figure S4B). Furthermore, when HKCA-trained PBMCs, after a 5-day rest, were restimulated with LPS with or without chloroquine and hydroxychloroquine treatment, we also observed no suppression of cytokine production (Figure S4C). Together, these data indicate that chloroquine and hydroxychloroquine specifically affect trained immunity, and that this is not a general inhibitory effect on cytokine production.

We subsequently examined if chloroquine and hydroxychloroquine affect trained immunity-mediated activity in the context of viral infection. HKCA-trained PBMCs were, after a 5-day rest, restimulated with a virus-like stimulus (poly I:C). We observed enhanced cytokine production in trained cells after restimulation with poly I:C, which could be mitigated by chloroquine and hydroxychloroquine treatment during the training stimulus (Figure 4D). These findings indicate that chloroquine and hydroxychloroquine hamper the boosting effect of trained immunity on the innate immune response against viral stimuli.

Because IFNs play a central role in viral immune responses, and our monocyte transcriptome data from COVID-19 patients revealed enhanced IFN-stimulated gene expression, we investigated how inflammatory monocytes respond to restimulation with IFNα, IFNβ, and IFNγ. Interestingly, we observed an enhanced production of IL-6 and TNF-α. This effect was mitigated by chloroquine and hydroxychloroquine treatment during the HKCA training stimulus (Figure 4E). We sought to assess if this was mediated through altered lysosomal function. Lysosomal proteins function in an acidic environment with a pH of around 4.5 to 5.0, which is maintained by the vacuolar-type H^+^-ATPase (V-ATPase). V-ATPase activity is also required for mTOR activation. Pharmacologic blocking of V-ATPase with bafilomycin A1 prevented trained immunity, mirroring the effects of chloroquine and hydroxychloroquine, as well as that of mTOR inhibition (Figures 4F and 4G; Figures S4D and S4E).

Next, we investigated the transcriptomic effects of hydroxychloroquine treatment on trained monocytes. PBMCs were stimulated for 24 h with either RPMI, HKCA, or HKCA and hydroxychloroquine, after which we purified monocytes and performed RNA sequencing. Hydroxychloroquine treatment significantly altered the monocyte transcriptome. Interestingly, hydroxychloroquine prevented the enhanced expression of genes that encode IL-1α and IL-1β, which play central roles in trained immunity (Figure 4H). Pathway analysis of differentially expressed genes revealed that hydroxychloroquine treatment substantially downregulated genes, including IFN-stimulated genes, related to inflammatory responses (Figure 4I). We also observed distinct RNA expression patterns in metabolic pathways important for inflammation, namely those related to oxidative phosphorylation and cholesterol homeostasis (Figure 4I). Altogether, these data indicate that hydroxychloroquine prevents the induction of trained immunity and suppresses the expression of IFN-stimulated genes.

### Hydroxychloroquine Affects the Cellular Lipidome

**Figure 5:**
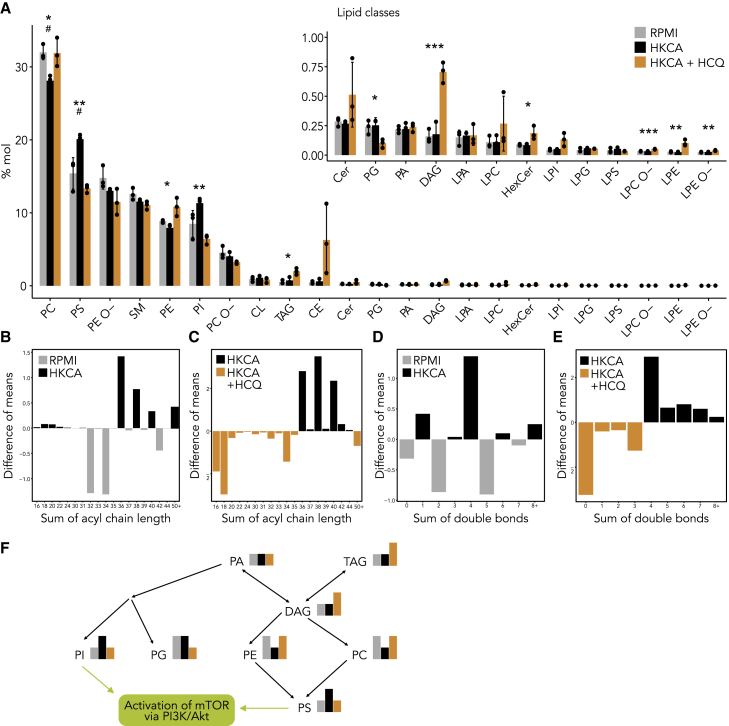
HCQ Affects the Monocyte Lipidome (A–E) PBMCs were stimulated with HKCA, HKCA+HCQ, or RPMI as a control for 24 h. Subsequently, monocytes were purified and analyzed for their lipid content using mass-spectrometry-based shotgun lipidomics (n = 3 per treatment group). (A) Abundance of lipid classes as the molar percentage of all lipids per treatment group. HKCA alone induced a significant decrease in phosphatidylcholines and an increase in phosphatidylserines compared to control cells, whereas HCQ induced significant changes in multiple lipid classes compared to HKCA-treated cells. The inserted graph shows low-abundant lipids on a smaller scale. (B and C) Analysis of acyl chain length of all lipids identified. HKCA training resulted in lipids with longer acyl chains than those of the control. HCQ induced even shorter acyl chains than the control. (D and E) Analysis of double bonds in all lipids identified. HKCA training did not cause marked effects compared to control cells. HCQ-treated cells contained more lipids with fewer double bonds than HKCA-trained cells. (F) Schematic representation of lipid metabolism showing lipid classes that are affected by either HKCA or HCQ treatment. Data are presented as mean ± SEM; ^#^p < 0.05 between control and HKCA; *p < 0.05, ∗∗p < 0.01, ∗∗∗p < 0.001 between HKCA and HKCA+HCQ; one-way ANOVA with Tukey post-test.

Our transcriptome data indicate that genes related to lipid metabolism play an important role in how hydroxychloroquine treatment prevents trained immunity. This corroborates previous studies in which the cholesterol synthesis pathway was shown to be involved in trained immunity.^25^^,^^27^ We were interested in the monocyte lipidome in the context of trained immunity and the effect of hydroxychloroquine on this process. Accordingly, we analyzed the monocyte lipidome after 24 h of HKCA stimulation with or without hydroxychloroquine by performing quantitative shotgun lipidomics, an unbiased mass-spectrometry-based method that can detect hundreds of lipid types present in cells.^28^ Principal-component analysis of the lipidomic data showed marked differences in the lipidomes of the HKCA-trained monocytes compared to trained monocytes treated with hydroxychloroquine and control monocytes (Figure S5A). Training with HKCA affected the concentrations of phosphatidylcholines (PCs) and phosphatidylserines (PSs) compared to control cells. Compared to HKCA training, hydroxychloroquine treatment altered a wide range of lipid classes, namely diacylglycerols (DAGs), hexosylceramides (HexCers), alkyl-ether-linked lyso-PCs (LPC O-), lyso-phosphatidylethanolamines (LPEs), alkyl-ether-linked lyso-phosphatidylethanolamines (LPE O-), PCs, PEs, phosphatidylglycerols (PGs), phosphatidylinositols (PIs), PSs, and triacylglycerols (TAGs) (Figure 5A). Interestingly, we observed that effects of HKCA training on various subspecies of PCs, PEs, PIs, and PSs could be prevented by treatment with hydroxychloroquine (Figures S5B–S5E). Concentrations of PE and PC, which both can be synthetized from DAG, were reduced in HKCA-trained cells and remained at the level of control cells with hydroxychloroquine treatment (Figure 5F). PS, which can be synthesized from PE and PC, was increased upon HKCA training and remained at the level of control values with hydroxychloroquine treatment (Figure 5F). A similar pattern could be observed for PI (Figure 5F). In addition to the quantitative changes, lipid configurations were altered (Figure 5B–5E). Lipids isolated from HKCA-trained cells had longer acyl chains than lipids from hydroxychloroquine-treated and control cells (Figures 5B and 5C), whereas no change in acyl chain length could be observed (Figure 5D). HKCA-trained cells treated with hydroxychloroquine contained more lipids with saturated acyl chains (none, one, or two double bonds) than HKCA-trained cells (Figure 5E).

Our data indicate that trained immunity is accompanied by profound changes in the lipidome of monocytes. These changes may affect both cell and organelle membranes, thereby attenuating the function and activation of membrane-bound proteins. In this respect, it is interesting to note that PS and PI are essential to the activation of the phosphoinositide 3-kinase (PI3K)/AKT kinase complex, an important activation step in the mTOR pathway, and that mTOR itself requires the lysosomal membrane for its activation (Figure 4A).^29^, ^30^, ^31^

### Hydroxychloroquine Prevents the Epigenetic Modifications Necessary to Induce Trained Immunity

**Figure 6:**
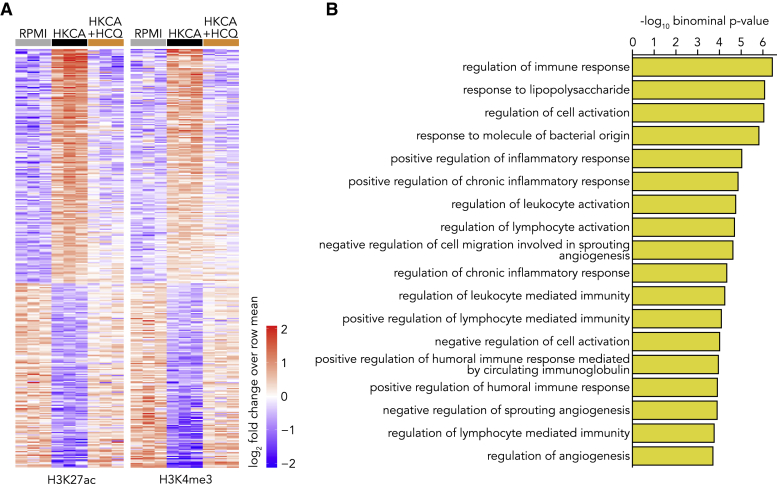
HCQ Prevents the Epigenetic Changes That Underly Trained Immunity (A and B) PBMCs were stimulated with HKCA, HKCA+HCQ, or RPMI as a control for 24 h, after which cells were rested for 5 days. Subsequently, monocytes were purified and ChIP was performed for H3K27ac and H3K4me3 (n = 3 per treatment group). (A) Heatmap showing relative H3K27ac (left panel) and H3K4me3 (middle panel) dynamics at sites with a significant change in histone mark abundance (FDR, <0.01) between HKCA-stimulated and control monocytes. (B) GO enrichment analysis of regulatory elements that remain epigenetically activated in HKCA-trained monocytes compared to HKCA+HCQ-treated cells (FDR, <0.01). Gene sets significantly associated at both an FDR Q value of <0.05 and a binominal p value of <0.05 are shown.

Epigenetic changes provide the molecular substrate of trained immunity in monocytes and macrophages. As our functional assays showed that hydroxychloroquine prevents trained immunity, we investigated the impact of hydroxychloroquine on epigenetic regulation in monocytes. For this purpose, we performed a whole-genome assessment of the histone marks histone 3 lysine 27 acetylation (H3K27ac) and histone 3 lysine 4 trimethylation (H3K4me3) by chromatin immunoprecipitation sequencing (ChIP-seq) in control monocytes as well as HKCA-trained monocytes treated with or without hydroxychloroquine. Monocytes were trained as described previously, and after 5 days of rest, monocyte-derived-macrophages were harvested for ChIP-seq. Epigenetic analysis by ChIP-seq of H3K27ac and H3K4me3 revealed marked differences between control and trained macrophages. After training and 5 days of resting, we found 352 peaks that had retained a significant change in the H3K27ac and H3K4me3 dynamic between HKCA and control cells, indicating that an epigenetic memory was established. Interestingly, all HKCA-induced changes could be prevented with hydroxychloroquine treatment (Figure 6A). Pathway analysis of differentially regulated peaks that remained active in HKCA-trained cells and were shut down in HKCA- and hydroxychloroquine-treated cells revealed pathways associated with immune responses and inflammation (Figure 6B). These data therefore confirm our functional assays and show that hydroxychloroquine treatment effectively prevents the epigenetic changes underlying HKCA-induced training and that this especially involves the regulation of inflammation-related genes.

## Discussion

SARS-CoV-2 infection primarily affects the upper respiratory tract and lung tissue. In most patients, an adequate immune response resolves the infection without causing organ damage. However, if the immune response is inadequate and viral clearance is impaired, severe pneumonia and other organ damage can develop, as is observed in patients with severe COVID-19.^1^ Here, we investigated the immune response in patients with COVID-19 and the immune-modulating action of hydroxychloroquine. We found that circulating monocytes from COVID-19 patients exhibit a phenotype of enhanced activation. Increased expression of IFN-stimulated genes by these cells associated with the development of more severe illness. We discovered that hydroxychloroquine can avert the H3K27 and H3K4 histone modifications that underly trained immunity, possibly through changes in the cellular lipidome. Trained immunity comprises a functional adaptation of monocytes that enhances their immunologic potential. Our findings provide insight into how hydroxychloroquine suppresses the trained innate immune response, including to virus-like stimuli and IFNs.

Hydroxychloroquine’s immunomodulatory effects have been known for decades, and it is commonly used to prevent flares in rheumatic diseases, like systemic lupus erythematous and rheumatoid arthritis.^10^^,^^32^^,^^33^ This drug can inhibit the production of cytokines, like IL-1β, IL-6, and TNF-α, by innate immune cells.^34^^,^^35^ Yet, the mechanism by which it inhibits cytokine production remains poorly understood. Hydroxychloroquine has a basic side chain and accumulates in the lysosome, where it exerts its effect, likely by impairing lysosome acidification rather than by targeting specific proteins. Previous studies support that hydroxychloroquine interferes with lysosomal processes, including autophagy,^36^ antigen processing, and major histocompatibility complex class II presentation,^37^^,^^38^ as well as TLR7 and TLR9 processing and binding.^39^

We discovered a previously unknown immunomodulatory mechanism of hydroxychloroquine, namely that it prevents trained immunity through epigenetic modulation. This likely relates to lysosome acidification, as bafilomycin A has a similar effect. We hypothesize that hydroxychloroquine may prevent trained immunity through effects on mTOR signaling because mTOR closely interacts with the lysosome and is activated on its surface. Metabolic information from the lysosome is transmitted to the cell primarily through mTOR signaling,^40^ which is key to mediating inflammation. A previous report demonstrated hydroxychloroquine’s effect on the mTOR pathway by showing that hydroxychloroquine decreased cellular levels of phospho-S6, a readout for mTOR activity.^41^ Interestingly, hydroxychloroquine also has marked effects on lipid metabolism in monocytes. Changes in the expression of genes involved in lipid metabolism were previously found to play an important role in trained immunity.^25^ Our lipidomic studies showed that lipids belonging to the PI and PS class are upregulated upon HKCA training and that hydroxychloroquine treatment could prevent this increase. It is worth noting that mTOR activation by the PI3K/AKT pathway depends on the action of PS,^31^ which brings AKT to the plasma membrane where it can be activated by PIs.^30^ Further studies are required to unravel the interaction between the lipidome and mTOR signaling in the context of trained immunity.

Chloroquine and hydroxychloroquine use for COVID-19 remains a topic of intense debate and investigation. The recently published Recovery and Solidarity trials found no beneficial effects of hydroxychloroquine in hospitalized COVID-19 patients. In fact, the Recovery trial found that hydroxychloroquine-treated patients were more likely to require invasive mechanical ventilation or die.^42^^,^^43^ Currently, 114 randomized controlled trials are recruiting patients to investigate these drugs for the prevention or treatment of COVID-19 (https://clinicaltrials.gov). Especially in the context of their use as a prophylaxis, important efforts are being made to investigate their efficacy. The first randomized controlled trial on this topic, which included 821 patients, showed that hydroxychloroquine as a post-exposure prophylaxis did not prevent symptomatic SARS-CoV-2 infection.^12^ A subsequent study in 132 hospital-based health care workers found that use of this drug as a pre-exposure prophylaxis had no effect on the SARS-CoV-2 infection rate.^44^ Other trials are ongoing, among which is a large global trial that is recruiting over 40,000 health care workers to determine whether chloroquine or hydroxychloroquine are effective in preventing COVID-19 (https://copcov.org). Our findings provide mechanistic insights that shed new light on the usefulness of chloroquine and hydroxychloroquine in COVID-19. We show that these drugs prevent monocytes from adopting a trained immunity phenotype through effects on epigenetic reprogramming. Trained immunity is known to enhance the innate immune response and thereby facilitates the defense against infections. Previous studies have shown that the induction of trained immunity, e.g., through bacillus Calmette-Guérin (BCG) vaccination, can help prevent bacterial as well as viral infections.^45^^,^^46^ The fact that chloroquine and hydroxychloroquine avert trained immunity suggests that these drugs may not be beneficial for clearing viral infections like SARS-CoV-2 and argues against their use as a prophylactic for COVID-19. The question arises if the opposite, namely the induction of trained immunity, may actually be beneficial for preventing COVID-19. A randomized clinical trial is currently being conducted to investigate this question.^21^ It is interesting to note that a recent phase III randomized clinical trial showed that the induction of trained immunity by BCG vaccination prevents respiratory tract infections by 79%.^22^

Our findings also have potential relevance to other diseases like systemic lupus erythematosus and systemic sclerosis. Experimental studies have shown that trained immunity plays a role in the pathogenesis of these conditions.^47^^,^^48^ In this context, it is interesting to note that hydroxychloroquine is used to prevent, rather than treat, flare-ups of these auto-immune diseases. An inhibitory effect on trained immunity could provide a possible explanation for this preventive effect.

In summary, we found that hydroxychloroquine averts the induction of trained immunity in monocytes through epigenetic reprograming, namely by suppressing H3K27 acetylation and H3K4 histone trimethylation. This occurred concomitantly with changes in the cellular lipidome and decreased expression of IFN-stimulated genes. Trained immunity comprises a functional adaptation that enhances the potential of the innate immune system. Our findings provide mechanistic insight into how hydroxychloroquine suppresses the trained innate immune response, including virus-like stimuli and IFNs.

### Limitations of Study

We investigated the immune response in patients hospitalized for COVID-19. Our functional, flow cytometry, and RNA sequencing studies provide a detailed view of the innate immune response in these patients. However, a limitation is that we investigated a small sample size of 13 patients that harbors the potential of type II statistical error. Furthermore, our patients were all hospitalized and therefore comprise a subset of patients with severe disease, which prohibits extrapolation of our results to those with mild disease. We observed elevated cytokine responses to *ex vivo* stimulation of PBMCs from COVID-19 patients that is indicative of innate immune reprograming. However, we cannot be sure of the underlying molecular mechanism of this hyperresponsiveness because we did not perform whole-genome assessment of histone modifications in monocytes of these patients.

Regarding our results on the effect of hydroxychloroquine on trained immunity, it is important to note that we investigated this *in vitro* in a model in which human primary monocytes of healthy blood donors were trained with HKCA. In this *in vitro* model, hydroxychloroquine potently suppressed trained immunity. The dose we used in our experiment was based on the expected accumulation of hydroxychloroquine in monocytes at a dose generally administered to patients. French et al.^49^ showed that *in vitro*, 100 μM hydroxychloroquine generates intracellular levels similar to those in patients receiving therapy with 400 mg hydroxychloroquine daily. Nonetheless, further studies are required to investigate if hydroxychloroquine can suppresses trained immunity *in vivo*.

## STAR★Methods

### Key Resources Table

**Table:** 

REAGENT or RESOURCE	SOURCE	IDENTIFIER
**Antibodies**		

Rabbit polyclonal anti H3K4me3	Diagenode	Cat#pab-003-050; RRID: AB_2616052
Rabbit polyclonal anti-H3K27Ac	Diagenode	Cat#pab-196-050; RRID: AB_2637079
Anti-CD16 FITC	eBioscience	Cat#11-0168-42; RRID: AB_10805747
Anti-HLA-DR PE	Coulter	Cat#IM1639; RRID: AB_131284
Anti-CD14 PC7	eBioscience	Cat#25-0149; RRID: AB_1582277
Anti-CD56 APC	Coulter	Cat#IM2474; RRID: AB_130791
Anti-CD3 APC-750	Coulter	Cat#A94680; RRID: AB_2876783
Anti-CCR2 BV421	Beckton Dickinson	Cat#564067; RRID: AB_2738573
Anti-CD11b BV785	Biolegend	Cat#301346; RRID: AB_2563794
Live/Dead stain FVS620	Beckton Dickinson	Cat#564996; RRID: AB_2869636
Anti-CD19 APC-R700	Beckton Dickinson	Cat#564978; RRID: AB_2744308
Anti-CX3CR1 BV650	Biolegend	Cat#341625; RRID: AB_2716244
Anti-CD45 BV510	Biolegend	Cat#304035; RRID: AB_2561383

**Bacterial or Virus strain**		

Heat-killed SARS-CoV2	Isolated from patient	N/A
Biological Samples		
COVID-19 patient samples	This paper	N/A
Human PBMCs from buffy coats	Sanquin bloodbank	Cat#B2825R00

**Chemicals, Peptide and Recombinant Proteins**		

Ficoll-Paque (Lymphoprep)	StemCell Technologies, Inc.	Cat#07861
Glutamax	Thermo Fisher Scientific	Cat#35050
Pyruvate	Thermo Fisher Scientific	Cat#11360
Penicillin/Streptomycin	Thermo Fisher Scientific	Cat#15140
RPMI	Thermo Fisher Scientific	Cat#22409
EDTA	Sigma	Cat#E5134
DNase I	QIAGEN	Cat#79254
Chloroquine diphosphate	Sigma	Cat#C6628
Hydroxychloroquine sulfate	Sigma	Cat#H0915
Rapamycin (Sirolimus)	Selleckchem	Cat#S1039
Heat-killed Candida albicans	Invivogen	Cat#tlrl-hkca
Lipopolysaccharide (E.coli, O55:B5)	Sigma	Cat#L6529
Pam3CSK4	Invivogen	Cat#tlrl-pms
R848	Invivogen	Cat#tlrl-R848
Heat-killed staphylococcus aureus	ATCC	Cat#25923
Heat-killed streptococcus pneumoniae	ATCC	Cat#49619
16% Formaldehyde	Sigma	Cat#28908
Protease Inhibitor Cocktail (tablets)	Roche	Cat#04693132001
Phenylmethylsulfonyl fluoride (PMSF)	Roche	Cat#11359061001
NextFlex DNA barcodes	Bioo Scientific	N/A
NEXTflex adaptor stock	Bioo Scientific	N/A
Recombinant human IFNγ	Invivogen	Cat#rcyec-hinfg
polyI:C	Invivogen	Cat#tlrl-pic
Recombinant human IFNα	Invivogen	Cat#tlrl-hinfa8
Recombinant human IFNβ	R&D systems	Cat#8499-IF-010

**Critical Commercial Assays**		

MACS Pan Monocyte isolation kit	Miltenyi Biotech	Cat#130-096-537
Human IL-6 ELISA	R&D systems	Cat#DY206
Human TNFα ELISA	R&D systems	Cat#DY210
Human IFNγ ELISA	R&D systems	Cat#DY285B
Human IL-22 ELISA	R&D systems	Cat#DY782
Human IL-17 ELISA	R&D systems	Cat#DY317
Human Lactate assay	Biovision	Cat#K607
RNeasy Mini kit	QIAGEN	Cat#74106
MagnaChIP kit	Merck-Millipore	Cat#17-408
KAPA RNA HyperPrep Kit (with RiboErase)	KAPA Biosystems	Cat#08098140702
High Sensitivity DNA bioanalyzer kit	Agilent Technologies	Cat#5067-4626
dsDNA High Sensitivity Assay	Denovix	N/A
Kapa Hyper Prep Kit	KAPA Biosystems	Cat#7962363001

**Deposited Data**		

*In vitro* trained monocyte ChIP-seq and RNA-seq data	This paper	GEO: GSE159678
COVID-19 patient RNA-seq data	This paper	GEO: GSE159678

**Software and Algorithms**		

GraphPad Prism	Graphpad software	N/A
LipotypeZoom	Lipotype GmbH	N/A
R	R Core Team^50^	https://www.r-project.org/
Hisat	Kim et al.^51^	http://www.ccb.jhu.edu/software/hisat/index.shtml
Samtools	Li et al.^52^	http://samtools.sourceforge.net
DESeq2	Love at al.^53^	http://www.bioconductor.org/packages/release/bioc/html/DESeq2.html
ggplot2	Wickham^54^	https://ggplot2.tidyverse.org/
Complex Heatmaps	Gu et al.^55^	http://www.bioconductor.org/packages/devel/bioc/html/ComplexHeatmap.html
fgsea R package	Subramanian et al.^56^	https://bioconductor.org/packages/release/bioc/html/fgsea.html
Burros Wheeler Aligner	Li and Durbin^57^	http://maq.sourceforge.net/
Model-based analysis of ChIP-Seq	Zhang et al.^58^	https://github.com/macs3-project/MACS
BEDtools	Quinlan and Hall^59^	https://code.google.com/p/bedtools
GREAT	McLean et al.^60^	http://great.stanford.edu/public/html/

### Resource Availability

#### Lead Contact

Further information and requests for resources and reagents should be directed to and will be fulfilled by the Lead Contact, Raphaël Duivenvoorden (Raphael.Duivenvoorden@radboudumc.nl)

#### Materials Availability

This study did not generate new unique items.

#### Data and Code Availability

The RNA-seq and ChIP-seq datasets generated during this study are available at GEO: GSE159678 https://www.ncbi.nlm.nih.gov/geo/query/acc.cgi?acc=GSE159678

### Experimental Model and Subject Details

#### Human Subjects

For *in vitro* studies on human PBMCs and monocytes, buffy coats from healthy donors were obtained from Sanquin blood bank, Nijmegen after written informed consent, from which no additional details are available. Blood from COVID-19 patients was collected after written informed consent at Radboudumc (Detailed information about study subjects are listed in Table S1). The study was approved by the local medical ethics committee of the Radboudumc under reference number: 2020-6359.

#### Human PBMC isolation

PBMCs were isolated by differential centrifugation over Ficoll-Paque (Lymphoprep, StemCell Technologies, Inc.). Cells were washed three times in PBS. PBMCs and monocytes were resuspended in RPMI culture medium supplemented with 2mM glutamax, 1mM pyruvate and penicillin/streptomycin (all from Thermo Fisher Scientific) and counted on a Casy counter. Cell counts of whole blood and isolated PBMCs were also analyzed using a sysmex XN-450 automated hematology analyzer (Sysmex).

### Method Details

#### Training and inhibition experiments

Human PBMCs were trained as described before. In short, 500.000 PBMCs were added into 96-well flat bottom plates. Cells were allowed to adhere for 1h at 37°C. Cells were washed three times with PBS prior to stimulations. After washing cells were incubated with culture medium only as negative control, or treated with 100 μM chloroquine (Sigma Aldrich), 100 μM hydroxychloroquine (Sigma Aldrich) or 0.01 μM rapamycin (Selckchem) for 1 hour at 37°C. The chloroquine and hydroxychloroquine dose were based on a study by French et al.^49^ who showed that *in vitro* 100 uM is necessary to generate intracellular levels similar to those in patients receiving therapy with hydroxychloroquine 400 mg daily.^49^ Subsequently cells were incubated with 10^5^ cells/ml HKCA (Invivogen) for 24 hours together with the respective treatment for 24 hours at 37°C. Subsequently, cells were washed and cells were rested for five days in RPMI culture medium containing 10% FBS. After the resting period cells were stimulated with either RPMI as negative control, 10ng/ml LPS (Sigma Aldrich) 1ug/ml Pam3CSK4 (Invivogen), 10ug/ml polyI:C (Invivogen), 10ng/ml IFNα (Invivogen), 10ng/ml IFNβ (R&D systems) or 100ng/ml IFNγ (Invivogen). Where indicated hydroxychloroquine and chloroquine were added 1 hour prior to restimulation and 24 hours during restimulation.

#### PBMC stimulation of COVID-19 patients

PBMCs from COVID-19 patients were stimulated using 10 ng/ml LPS, 1 μg/ml Pam3CSK4, 10^6^ cells/ml HKCA and 10 μg/ml R848 (Invivogen) for 24 hours in RPMI without serum or with RPMI only as negative control or 10^6^ cells/ml heat-killed S*taphylococcus aureus* (ATCC) for 7 days in RPMI with 10% serum in 96-well round-bottom plates (Corning).

#### Monocyte isolation

Monocytes were isolated using negative MACS isolation with the Pan monocyte isolation kit (Miltenyi Biotech). Briefly, stimulated PBMCs were washed with PBS and incubated with versene solution (0.48mM EDTA, Sigma Aldrich) for 30 minutes at 37°C. Cells were scraped from the plates, counted, spun down and resuspended in MACS isolation buffer (PBS with 0.5% BSA and 2mM EDTA).

Monocytes from COVID-19 patients were isolated directly after isolation of PBMCs. PBMCs were counted, spun down and resuspended in MACS isolation buffer. Monocyte isolation was performed according to manufacturer’s instructions.

#### Cytokine measurements

Cytokine production was measured in supernatants using commercial ELISA kits for human TNFα, IL-6, IFNγ, IL-22 and IL-17 (R&D systems) according to manufacturer’s instruction.

#### Flow cytometry

Circulating immune cells and monocyte (sub)populations were identified by their expression markers using a CYTOflex flow cytometer (Beckman Coulter) (gating strategy in Figure S1C). Antibodies and dilutions used are CD45-BV510 (Biolegend, 1:100), CD14-PC7 (eBioscience, 1:100), CD16-FITC (eBioscience, 1:100), CD3-APC750 (Beckman Coulter, 1:50), CD19-APC-R700 (Becton Dickinson, 1:100), CD56-APC (Beckman Coulter, 1:50), HLA-DR-PE (Beckman Coulter, 1:20), CD11b-BV785 (Biolegend, 1:100), CCR2-BV421 (Becton Dickinson, 1:50), CX3CR1-BV650 (Biolegend, 1:50) and Live/Dead FVS620 (Becton Dickinson, according to manufacturers’ instructions). 500.000 PBMCs were stained with FVS620, subsequently underwent Fc blocking using 10% Heat-Inactivated human serum and were stained with antibodies in presence of Brilliant Stain buffer (Becton Dickinson) as multiple BV antibodies were used. Flow cytometry standards (FCS) files underwent pre-processing to remove debris, dead cells and doublets. Live single cells were then analyzed by both unsupervised computational analysis as well as manual gating in parallel. Characterization of monocytes subsets is according to current recommendations (See Figure S1C).^61^ For unsupervised computation analysis, FCS files were randomly down sampled to 20,000 events of the pre-processed files and subsequently concatenated to a single file containing all events. Controls and patients were labeled accordingly to be able to separate them after analysis. Unsupervised clustering was performed on the expression values of all markers using the tSNE plugin in FlowJo (Becton Dickinson, version 10.6.2), using 1000 iterations and a perplexity of 30. Manual gating of known cell populations (see gating strategy, Figure S1C) was used to identify populations and to check separation quality of the unsupervised clustering. The contribution of the control and patient populations to the total tSNE was then analyzed by separating the groups. Visual differences were then confirmed by manual gating and statistical analysis.

#### RNA isolation, library preparation and sequencing for transcriptomic analysis

For RNA isolation 1∗10^6^ isolated monocytes were resuspended in 350 μL of RNA later Buffer (QIAGEN). RNA was isolated using RNeasy kit (QIAGEN) including DNaseI (QIAGEN) digestions.

Total RNA isolated from monocytes was used for the preparation of the RNA sequencing libraries using the KAPA RNA HyperPrep Kit with RiboErase (KAPA Biosystems). In short, oligo hybridization and rRNA depletion, rRNA depletion cleanup, DNase digestion, DNase digestion cleanup, and RNA elution were performed according to protocol. Fragmentation and priming were performed at 94°C for 6 min. First strand synthesis, second strand synthesis and A-tailing was performed according to protocol. For the adaptor ligation, a 1.5 μM stock was used (NextFlex DNA barcodes, Bioo Scientific). First and second post-ligation cleanup was performed according protocol. A total of 11 PCR cycles were performed for library amplification. The library amplification cleanup was done using a 0.8x followed by a 1.0x bead-based cleanup. Library size was determined using the High Sensitivity DNA bioanalyzer kit, and the library concentration was measured using the dsDNA High Sensitivity Assay (Denovix). Paired-end sequencing reads of 50 bp were generated using an Illumina NextSeq 500.

#### Preparation of samples and lipid extraction for mass spectrometry lipidomics

For lipidomic analysis 10∗10^6^ isolated monocytes were collected in to microcentrifuge tubes, centrifuged at 1000*x*g for 5 minutes at 4°C. The supernatant was removed and cells were snap frozen in liquid nitrogen. Mass spectrometry-based lipid analysis was performed at Lipotype GmbH (Dresden, Germany) as described in Sampaio et al.^28^ Lipids were extracted using a two-step chloroform/methanol procedure.^62^ Samples were spiked with internal lipid standard mixture containing: cardiolipin 16:1/15:0/15:0/15:0 (CL), ceramide 18:1;2/17:0 (Cer), diacylglycerol 17:0/17:0 (DAG), hexosylceramide 18:1;2/12:0 (HexCer), lysophosphatidate 17:0 (LPA), lyso-phosphatidylcholine 12:0 (LPC), lyso-phosphatidylethanolamine 17:1 (LPE), lyso-phosphatidylglycerol 17:1 (LPG), lyso-phosphatidylinositol 17:1 (LPI), lyso-phosphatidylserine 17:1 (LPS), phosphatidate 17:0/17:0 (PA), phosphatidylcholine 17:0/17:0 (PC), phosphatidylethanolamine 17:0/17:0 (PE), phosphatidylglycerol 17:0/17:0 (PG), phosphatidylinositol 16:0/16:0 (PI), phosphatidylserine 17:0/17:0 (PS), cholesterol ester 20:0 (CE), sphingomyelin 18:1;2/12:0;0 (SM) and triacylglycerol 17:0/17:0/17:0 (TAG). After extraction, the organic phase was transferred to an infusion plate and dried in a speed vacuum concentrator. 1st step dry extract was re-suspended in 7.5 mM ammonium acetate in chloroform/methanol/propanol (1:2:4, V:V:V) and 2nd step dry extract in 33% ethanol solution of methylamine in chloroform/methanol (0.003:5:1; V:V:V). All liquid handling steps were performed using Hamilton Robotics STARlet robotic platform with the Anti Droplet Control feature for organic solvents pipetting.

#### Mass spectrometry data acquisition

Samples were analyzed by direct infusion on a QExactive mass spectrometer (Thermo Scientific) equipped with a TriVersa NanoMate ion source (Advion Biosciences). Samples were analyzed in both positive and negative ion modes with a resolution of Rm/z = 200 = 280000 for MS and Rm/z = 200 = 17500 for MSMS experiments, in a single acquisition. MSMS was triggered by an inclusion list encompassing corresponding MS mass ranges scanned in 1 Da increments.^63^ Both MS and MSMS data were combined to monitor CE, DAG and TAG ions as ammonium adducts; PC, PC O-, as acetate adducts; and CL, PA, PE, PE O-, PG, PI and PS as deprotonated anions. MS only was used to monitor LPA, LPE, LPE O-, LPI and LPS as deprotonated anions; Cer, HexCer, SM, LPC and LPC O- as acetate adduct.

#### Chromatin Immunoprecipitation

Isolated monocytes were resuspended in RPMI culture medium and fixed using formaldehyde (1% final concentration, Sigma Aldrich) for 10 minutes at room temperature. Unreacted formaldehyde was quenched with 125 mM glycine and incubated for 5 minutes at room temperature. Cells were washed twice in PBS containing protease inhibitor cocktail (Roche) and 1 mM PMSF (Roche), and subsequently snap frozen in liquid nitrogen. Cell pellets were stored at −80°C for further use. Cells were sonicated at a concentration of 15 million cells/ml using a Bioruptor pico sonicator (Diagenode; 10 cycles, 30 s on, 30 s off, at 4°C). Immunoprecipitation was performed using the MagnaChIP kit (Merck Millipore) according to manufacturer’s instruction. In short, 500,000 cells were incubated overnight with 1 μg H3K4me3 or H3K27Ac antibody (Diagenode) and protein A magnetic beads at 4°C. Beads and chromatin/antibody mixture were washed four times for 5 minutes at 4°C. After washing chromatin was eluted and proteins were degraded using proteinase K. DNA was purified using spin columns and eluted in millliQ.

#### Library preparation and sequencing of ChIP samples

ChIP-seq libraries were prepared using the Kapa Hyper Prep Kit according to manufacturer’s protocol, with the following modifications. 2.5 μL of the NEXTflex adaptor stock (600 nM, Bioo Scientific) was used for adaptor ligation of each sample. Libraries were amplified with 12-15 PCR cycles followed by a double post-amplification clean-up was used to ensure proper removal of adapters. Samples were analyzed for purity using a High Sensitivity DNA Chip on a Bioanalyzer 2100 system (Agilent). Libraries were paired-end sequenced to a read length of 50 bp on an Illumina NextSeq500.

### Quantification and Statistical Analysis

#### *In vitro* experiments and flow cytometry data analysis

For *ex vivo* stimulations and flow cytometry data, data are shown as mean ± SEM and significance is tested using two-sided Student’s t test (for normally distributed data) or Kruskal Wallis. For *in vitro* trainings-experiments, data is shown as mean ± SEM and significance is tested with one-way ANOVA with Dunnett’s post-test. Data was analyzed using GraphPad Prism 5.0. P value of 0.05 was considered to be statistically significant.

#### RNA-seq data analysis

RNA-sequencing reads were aligned with Hisat2 version 2.0.4 to the provided and pre-indexed hg38 transcript assembly from UCSC, with alignment tailoring for transcript assemblers enabled.^51^ Samtools was used to filter reads with a quality score lower than 20, and PCR duplicates were removed with Picard.^52^ Reads per gene were counted with the htseq-count script from the Hisat2 software suite using the GTF file corresponding to the transcript assembly, with reverse strandness enabled and identification attribute set to gene_id. Differential gene expression analysis was performed with the DESeq2 package version 1.18.1.^53^ Genes with no reads mapped in any of the samples were filtered prior to differential expression analysis. The ‘rlogTransformation’ function in DESeq2 was used to normalize, transform and noise-stabilize the expression data for visualization purposes. All analyses were performed in R and figures were generated with the ggplot2 and ComplexHeatmap R packages.^50^^,^^54^^,^^55^

Gene set enrichment analysis was performed with the fgsea R package, by ranking genes with the ‘lfcShrink’ function in DESeq2.^53^^,^^56^ The Hallmark and Gene Ontology (GO) gene set databases from MSigDB v6.2 were tested for significant associations with prognosis or response.^56^ At least 1,000,000 permutations were performed to control for the false discovery rate, and the minimal and maximum size of gene sets to be considered for analysis were set to 15 and 500 genes, respectively. To prevent masking of potential heterogeneity within patient groups, gene set enrichment analysis results were visualized by plotting the mean gene expression change of all detected genes in a significant gene set for each sample separately.

#### ChIP-seq data analysis

ChIP sequencing data was aligned to human genome hg19 with BWA.^57^ Samtools was used to filter reads with a quality score lower than 20, and PCR duplicates were removed with Picard.^52^ Peaks were identified with MACS 2.2.6 in paired-end mode and ‘call-summits’ enabled at a false discovery rate of 0.01.^58^ A union of all identified peaks was generated with BEDTools, which was used to count reads per peak in each sample.^59^ Read counts were analyzed with DESeq2 to identify significant dynamics, as described for the RNA-seq analysis. We used GREAT to identify significantly associated gene ontologies, and to assign each ChIP peak to its closest gene for integration between ChIP- and RNA-seq data.^60^

#### Lipidomics data analysis

Data were analyzed with in-house developed lipid identification software based on LipidXplorer.^64^^,^^65^ Data post-processing and normalization were performed using an in-house developed data management system. Only lipid identifications with a signal-to-noise ratio > 5, and a signal intensity 5-fold higher than in corresponding blank samples were considered for further data analysis. Further data analysis was performed using a web-based analysis program lipotypeZoom. Figures were generated in R with the ggplot2 and ComplexHeatmap R packages.^50^^,^^54^^,^^55^ Data are shown as mean ± SEM, significance was determined using one-way ANOVA and Tukey post-test.

## Supplemental Information

Document S1. Figures S1–S5 and Tables S1–S4

Document S2. Article plus Supplemental Information
