# Supplementary material for: Hydroxychloroquine Inhibits the Trained Innate Immune Response to Interferons
Source: Cell Rep Med. 2020 Nov 10;1(9):100146. doi: 10.1016/j.xcrm.2020.100146 (PMC7762774; doi:10.1016/j.xcrm.2020.100146)
Supplement: Document S1. Figures S1–S5 and Tables S1–S4 [file mmc1.pdf]

Cell Reports Medicine, Volume *1*

Supplemental Information

Hydroxychloroquine Inhibits the Trained

Innate Immune Response to Interferons

Nils Rother, Cansu Yanginlar, Rik G.H. Lindeboom, Siroon Bekkering, Mandy M.T. van Leent, Baranca Buijsers, Inge Jonkman, Mark de Graaf, Marijke Baltissen, Lieke A. Lamers, Niels P. Riksen, Zahi A. Fayad, Willem J.M. Mulder, Luuk B. Hilbrands, Leo A.B. Joosten, Mihai G. Netea, Michiel Vermeulen, Johan van der Vlag, and Raphaël Duivenvoorden

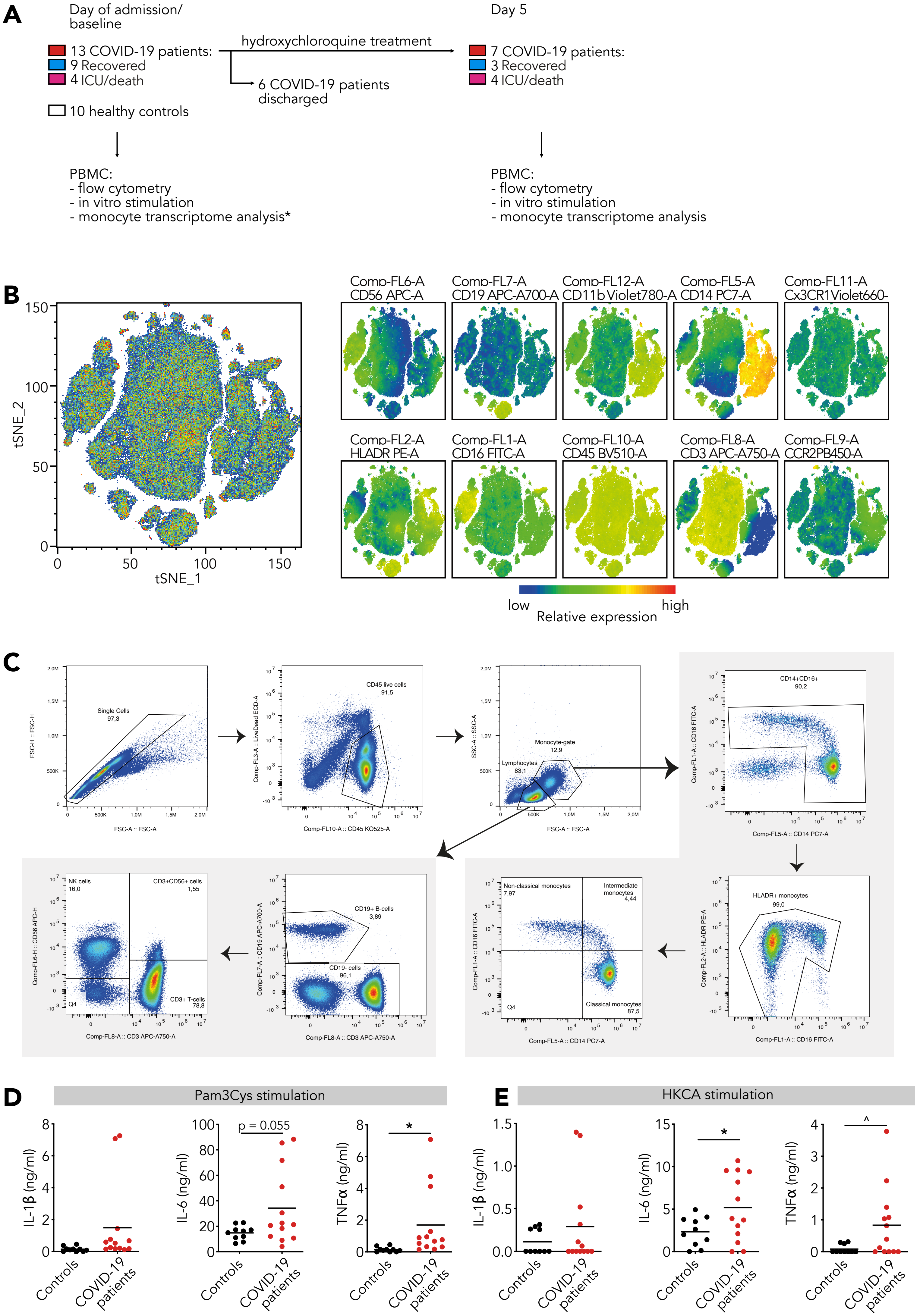
A
Day of admission/ baseline
13 COVID-19 patients:
9 Recovered
4 ICU/death
10 healthy controls
hydroxychloroquine treatment
6 COVID-19 patients discharged
Day 5
7 COVID-19 patients:
3 Recovered
4 ICU/death
PBMC:
- flow cytometry
- in vitro stimulation
- monocyte transcriptome analysis*
PBMC:
- flow cytometry
- in vitro stimulation
- monocyte transcriptome analysis
B
tSNE_1
tSNE_2
Comp-FL6-A CD56 APC-A
Comp-FL7-A CD19 APC-A700-A
Comp-FL12-A CD11b Violet780-A
Comp-FL5-A CD14 PC7-A
Comp-FL11-A Cx3CR1Violet660-
Comp-FL2-A HLADR PE-A
Comp-FL1-A CD16 FITC-A
Comp-FL10-A CD45 BV510-A
Comp-FL8-A CD3 APC-A750-A
Comp-FL9-A CCR2PB450-A
low
high
Relative expression
C
Single Cells 97,3
FSC-A :: FSC-A
FSC-H :: FSC-H
CD45 live cells 91,5
Comp-FL10-A :: CD45 KO525-A
Comp-FL3-A :: LiveDead ECD-A
Monocyte-gate 12,9
Lymphocytes 83,1
SSC-A :: SSC-A
CD14+CD16+ 90,2
Comp-FL5-A :: CD14 PC7-A
Comp-FL1-A :: CD16 FITC-A
HLADR+ monocytes 99,0
Comp-FL1-A :: CD16 FITC-A
Comp-FL2-A :: HLADR PE-A
Non-classical monocytes 7,97
Intermediate monocytes 4,44
Classical monocytes 87,5
Q4
Comp-FL5-A :: CD14 PC7-A
Comp-FL1-A :: CD16 FITC-A
CD19+ B-cells 3,89
CD19- cells 96,1
Comp-FL8-A :: CD3 APC-A750-A
Comp-FL7-A :: CD19 APC-A700-A
NK cells 16,0
CD3+CD56+ cells 1,55
CD3+ T-cells 78,8
Q4
Comp-FL8-A :: CD3 APC-A750-A
Comp-FL6-H :: CD56 APC-H
D
Pam3Cys stimulation
IL-1β (ng/ml)
IL-6 (ng/ml)
p = 0.055
TNFα (ng/ml)
*
Controls
COVID-19 patients
E
HKCA stimulation
IL-1β (ng/ml)
IL-6 (ng/ml)
*
TNFα (ng/ml)
^
Controls
COVID-19 patients

Supplementary Figure 1: Innate immune response in COVID-19 patients at the time of presentation. Related to Figure 1.
(A) Schematic representation of study outline involving COVID-19 patients. PBMCs of COVID-19 patients were isolated at admission (n=13) and five days later (n=7). PBMCs were used for flow cytometric analysis and *in vitro* stimulation, healthy control PBMCs (n=10) served as control. * Monocyte transcriptome analysis was performed for COVID-19 patients.

(B) Flow cytometry marker expression shown in tSNE plots in **Figure 1A** and **Figure 2A**.

(C) Flow cytometry gating strategy for quantifications of lymphocytes, monocytes and marker expression on monocytes.

(D and E) Isolated PBMCs were stimulated with Pam3CSK4 (D) or HKCA (E) for 24 hours after which production of IL-1β, IL-6 and TNFα was quantified in the supernatant using ELISA. COVID-19 patient PBMCs show increased cytokine production upon stimulation with either stimulus (n=13 for COVID-19 patients, n=10 for healthy controls).

Data are presented as mean ± SEM. ^$p<0.06$, *$p<0.05$ for two-sided student's t-test (for normally distributed data) or Kruskal-Wallis test.

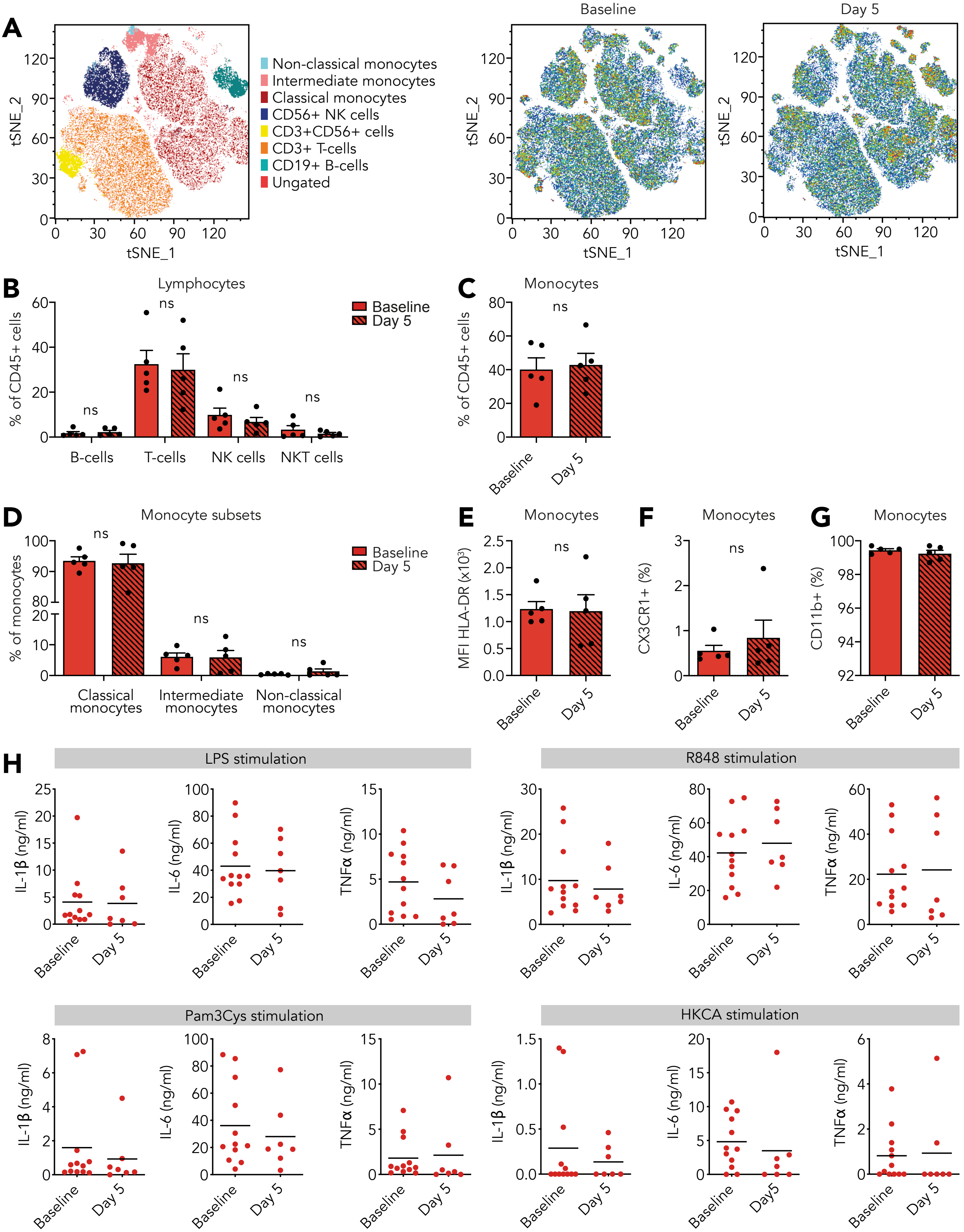

A
Non-classical monocytes
Intermediate monocytes
Classical monocytes
CD56+ NK cells
CD3+CD56+ cells
CD3+ T-cells
CD19+ B-cells
Ungated
tSNE_1
tSNE_2
Baseline
Day 5
B
Lymphocytes
% of CD45+ cells
ns
B-cells
T-cells
NK cells
NKT cells
C
Monocytes
D
Monocyte subsets
% of monocytes
Classical monocytes
Intermediate monocytes
Non-classical monocytes
E
MFI HLA-DR (x10³)
F
CX3CR1+ (%)
G
CD11b+ (%)
H
LPS stimulation
R848 stimulation
Pam3Cys stimulation
HKCA stimulation
IL-1β (ng/ml)
IL-6 (ng/ml)
TNFα (ng/ml)
Day5

Supplementary Figure 2: Innate immune response in COVID-19 patients at the time of presentation does not differ with response five days after admission. Related to Figure 2.
(A-G) PBMCs isolated from COVID-19 patients at admission and five days after admission were analyzed using flow cytometry (n=10 for COVID-19 patients at admission, n=5 for COVID-19 patients five days after admission).

(A) tSNE plots showing unsupervised clustering on the expression of 10 markers (CD45, CD14, CD16, CD3, CD19, CD56, HLA-DR, CD11b, CCR2 and CX3CR1) in COVID-19 patients at admission and five days after admission.

(B) Quantification of lymphocytes using gating strategy shown in **Supplementary Figure 1C** identified no difference between timepoints.

(C and D) Quantification of monocytes showed no difference between COVID-19 patients at admission and five days after admission.

(E-G) Analysis of marker expression of HLA-DR, CX3CR1 and CD11b on monocytes revealed no difference between COVID-19 patients at admission and five days after admission.

(H) Isolated PBMCs were stimulated with LPS, R848, Pam3CSK4 or HKCA for 24 hours after which production of IL-1β, IL-6 and TNFα was quantified in the supernatant using ELISA. COVID-19 patient PBMCs show no difference in cytokine production on different timepoints. (n=12 for COVID-19 patients at baseline, n=7 for COVID-19 patients five days after admission)

Data are presented as mean ± SEM. ^p<0.06, *p<0.05 for two-sided student's t-test (for normally distributed data) or Kruskal-Wallis test.

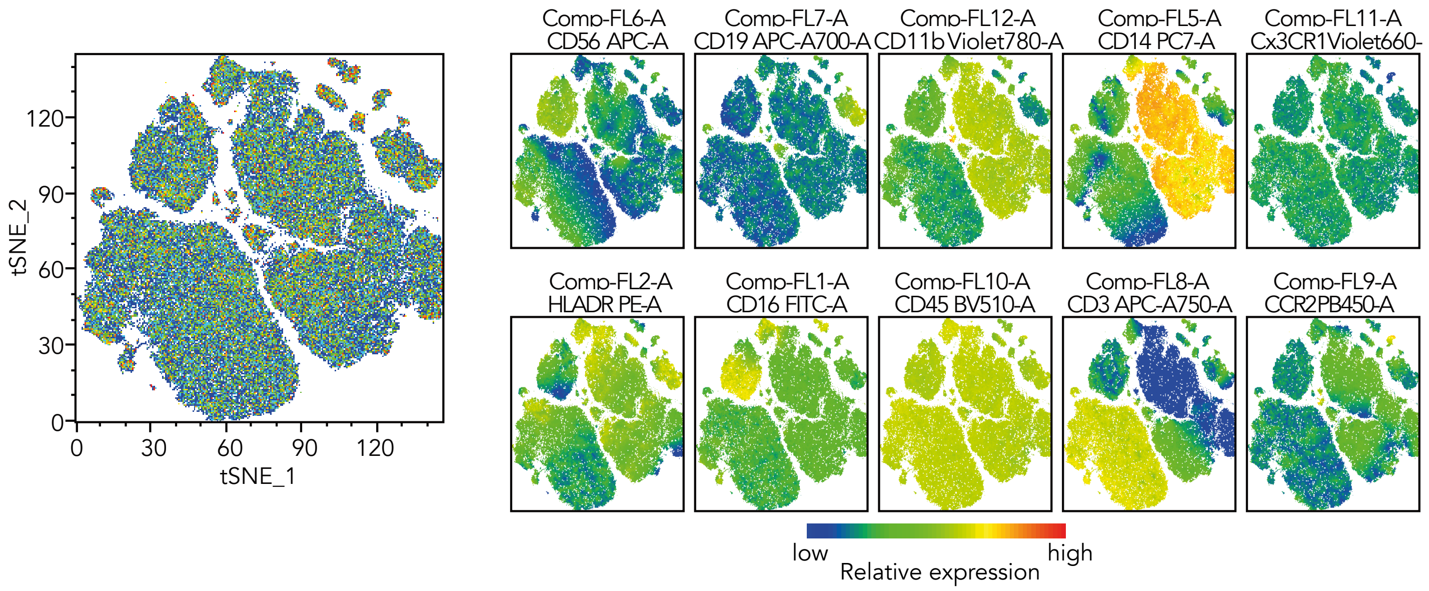

Supplementary Figure 3: Marker expression of tSNE plots. Related to Figure 3.

Flow cytometry marker expression shown in tSNE plots in **Figure 3A**.

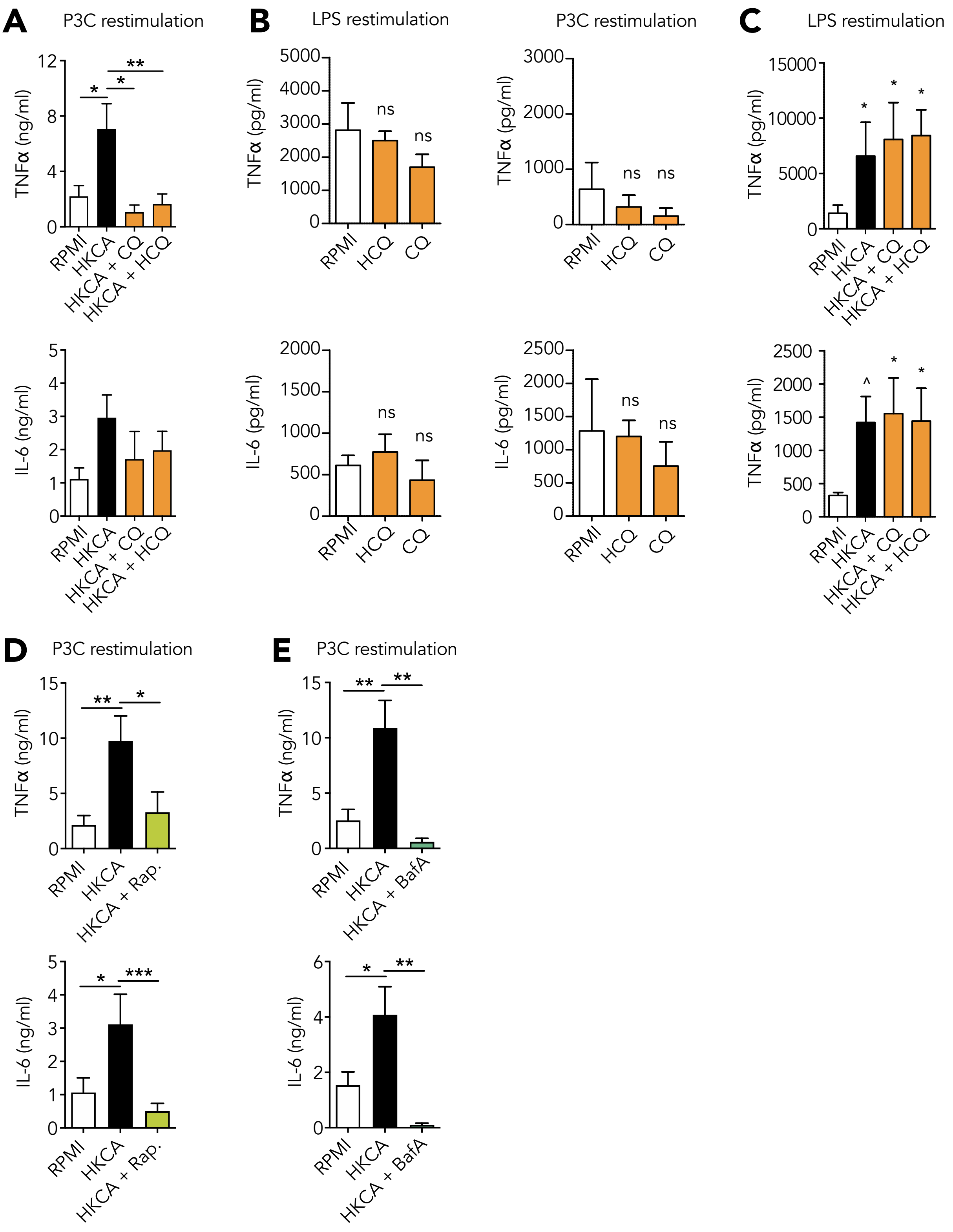

Supplementary Figure 4: Hydroxychloroquine prevents the induction of trained immunity. Related to Figure 4.

(A) IL-6 and TNFα production upon restimulation with Pam3CSK4 of HKCA-trained PBMCs treated with HCQ and CQ is abrogated (n=7-17)

(B) HCQ and CQ do not abrogate IL-6 and TNFα production in untrained PBMCs. (n=5)

(C) Hydroxychloroquine and chloroquine were added during restimulation after the resting period instead of during first 24 hours. Both, hydroxychloroquine and chloroquine do not reverse trained immune response when applied during restimulation (n=4, significance compared to RPMI).

(D and E) IL-6 and TNFα production upon restimulation with Pam3CSK4 of HKCA-trained PBMCs can be prevented using rapamycin (D, n=11) and Bafilomycin A (E, n=4-9).

Data are presented as mean ± SEM; *p<0.05, **p<0.01, ***p<0.001; One-way ANOVA with Dunnett's post-test.

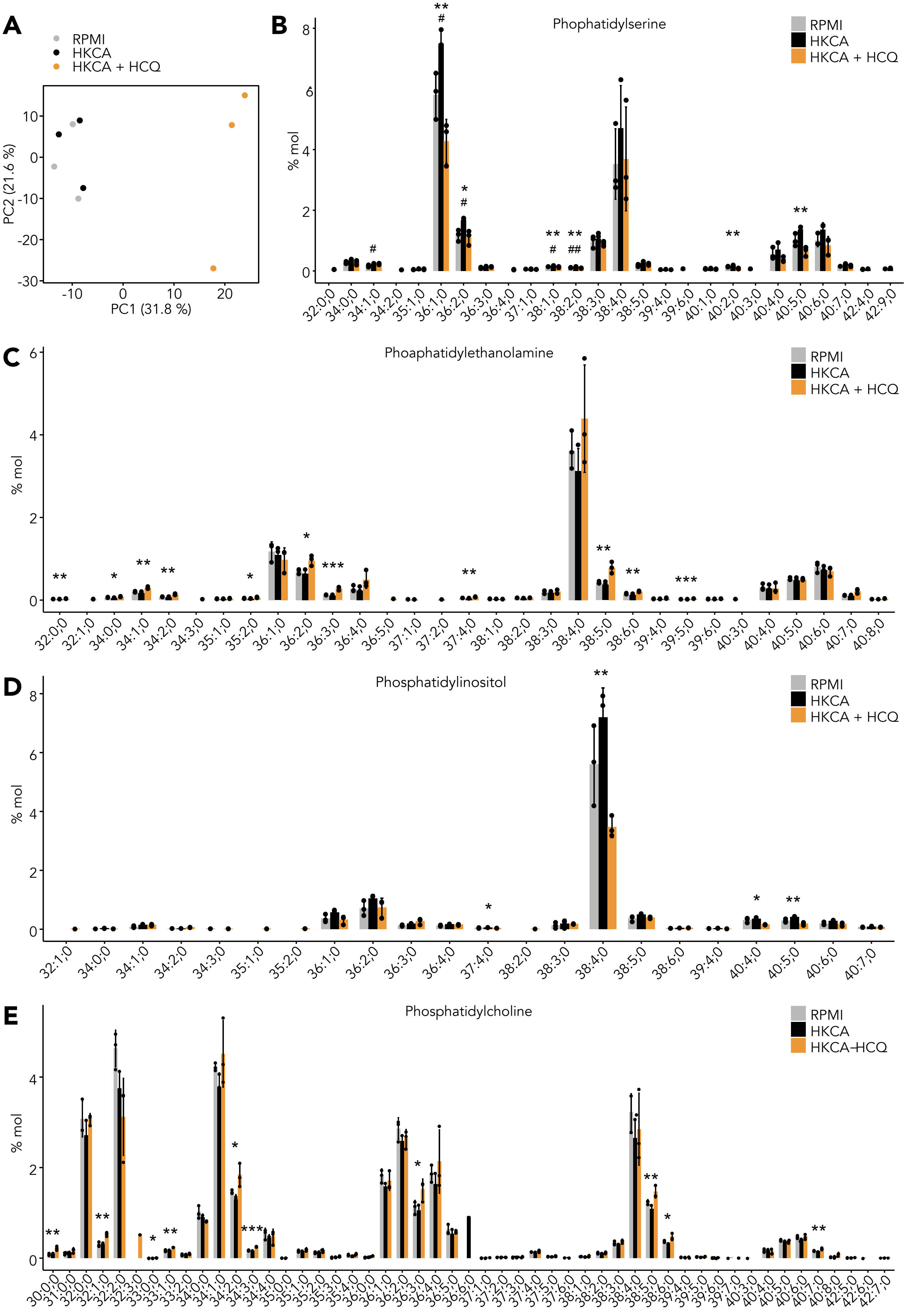
A
RPMI
HKCA
HKCA + HCQ
PC2 (21.6 %)
PC1 (31.8 %)
B
Phophatidylserine
RPMI
HKCA
HKCA + HCQ
% mol
32:0;0 34:0;0 34:1;0 34:2;0 35:1;0 36:1;0 36:2;0 36:3;0 36:4;0 37:1;0 38:1;0 38:2;0 38:3;0 38:4;0 38:5;0 39:4;0 39:6;0 40:1;0 40:2;0 40:3;0 40:4;0 40:5;0 40:6;0 40:7;0 42:4;0 42:9;0
C
Phoaphatidylethanolamine
RPMI
HKCA
HKCA + HCQ
% mol
32:0;0 32:1;0 34:0;0 34:1;0 34:2;0 34:3;0 35:1;0 35:2;0 36:1;0 36:2;0 36:3;0 36:4;0 36:5;0 37:1;0 37:2;0 37:4;0 38:1;0 38:2;0 38:3;0 38:4;0 38:5;0 38:6;0 39:4;0 39:5;0 39:6;0 40:3;0 40:4;0 40:5;0 40:6;0 40:7;0 40:8;0
D
Phosphatidylinositol
RPMI
HKCA
HKCA + HCQ
% mol
32:1;0 34:0;0 34:1;0 34:2;0 34:3;0 35:1;0 35:2;0 36:1;0 36:2;0 36:3;0 36:4;0 37:4;0 38:2;0 38:3;0 38:4;0 38:5;0 38:6;0 39:4;0 40:4;0 40:5;0 40:6;0 40:7;0
E
Phosphatidylcholine
RPMI
HKCA
HKCA+HCQ
% mol

Supplementary Figure 5: Lipidomic analysis of hydroxychloroquine treated monocytes. Related to Figure 5.

(A-E) PBMCs were stimulated with HKCA, HKCA+HCQ or RPMI as control for 24h. Subsequently monocytes were purified and analyzed for their lipid content using mass spectrometry-based shotgun lipidomics.

(A) PCA plot of all sample replicates. HCQ induces drastic changes in lipid abundance.

(B-E) Abundance of lipid subspecies of phosphatidylserines (B), phosphatidylethanolamines (C), phosphatidylinositols (D) and phosphatidylcholines (E) in control, HKCA-treated and HKCA+HCQ -treated cells are presented as molar percentage of all lipids per treatment group.

N=3 per treatment group. Data are presented as mean ± SEM; $^{\#}p<0.05$, $^{\#\#}p<0.01$, $^{\#\#\#}p<0.001$ between Control and HKCA; p<0.05, **p<0.01, ***p<0.001 between HKCA and HKCA+HCQ; One-way ANOVA with Tukey post-test

Supplementary Table 1: Demographics and characteristics of COVID-19 patients. Data is the median (inter quartile range) or number (no.) and percentage (%). Related to Figure 1.

		Disease outcome	
	**All patients**	**Recovered**	**ICU / Death**
**Characteristics at presentation**	**n=13**	**n=9**	**n=4**
Age, years	68 (54-73)	66 (52-73)	70 (55-79)
Male, no. (%)	11 (85%)	7 (78%)	4 (100%)
Medical history, no. (%)			
Pulmonary disease	5 (38%)	3 (33%)	2 (50%)
Cardiovascular disease	3 (23%)	2 (22%)	1 (25%)
Hypertension	4 (31%)	2 (22%)	2 (50%)
Diabetes	1 (8%)	0 (0%)	1 (25%)
Cancer	3 (23%)	3 (33%)	0 (0%)
Chronic kidney disease	3 (23%)	1 (11%)	2 (50%)
Vital signs			
Saturation, %	95.0 (94.5-97.0)	95.0 (95.0-97.5)	94.5 (93.3-95.8)
Oxygen therapy, L/min	2 (0-3)	2 (0-3)	1.5 (0-5.3)
Respiratory rate, /min	18 (16-25)	19 (17-26)	17 (15-21)
Pulse, /min	79.0 (67.5-96.5)	77.0 (63.5-96.5)	92.5 (78.5-105.8)
Systolic blood pressure, mmHg	139 (120-146)	139 (122-146)	130 (114-163)
Diastolic blood pressure, mmHg	77 (72-85)	77 (72-85)	79 (70-87)
Symptoms, no. (%)			
Fever	8 (61.5)	6 (66.7)	2 (50)
Dyspnea	7 (53.8)	4 (44.4)	3 (75)
Cough	10 (76.9)	8 (88.9)	2 (50)
Myalgia	2 (15.4)	2 (22.2)	0 (0)
Diarrhea	1 (7.7)	1 (11.1)	0 (0)
CT-thorax severity score	11 (8-13)	8 (7-13)	12 (11-12)
**Characteristics at day five of admission**			
Admitted at ICU, no. (%)	**n=7**	**n=3**	**n=4**
Requires oxygen therapy, no. (%)	2 (29%)	0 (0%)	2 (50%)
	4 (57%)	1 (33%)	3 (75%)

Supplementary Table 2: Whole blood and isolated PBMCs cell count of COVID-19 patients at presentation and five days after admission as measured using a sysmex XN-450 automated hematology analyzer. Data is the median (inter quartile range) or percentage (%). Related to Figure 1.

**Characteristics**	**All patients** N=13	**Outcome** **Recovered** N=9	**ICU / Death** N=4
Whole blood at presentation			
White blood cells (10^3/uL)	5.47 (4.04-7.81)	5.47 (4.28-7.52)	5.83 (3.98-9.15)
Neutrophils (%)	78.60 (60.25-84.55)	77.90 (60.25-84.55)	82.55 (62.00-89.90)
Lymphocytes (%)	12.70 (8.75-28.20)	13.90 (9.20-32.05)	10.90 (5.95-18.93)
Monocytes (%)	6.50 (4.00-10.85)	6.50 (4.90-10.60)	6.20 (2.98-11.83)
Eosinophils (%)	0.00 (0.00-0.50)	0.30 (0.00-0.50)	0.00 (0.00-7.65)
Basophils (%)	0.30 (0.20-0.45)	0.30 (0.20-0.40)	0.40 (0.23-0.50)
PBMC at presentation			
White blood cells (10^3/uL)	3.32 (2.58-6.43)	3.36 (3.06-9.02)	2.44 (1.83-4.14)
Neutrophils (%)	11.10 (5.70-17.05)	9.80 (4.55-21.30)	11.30 (7.50-19.00)
Lymphocytes (%)	62.30 (45.50-69.60)	62.30 (42.25-73.05)	59.25 (46.78-70.00)
Monocytes (%)	25.30 (19.55-38.70)	24.50 (19.55-40.15)	26.25 (19.08-38.75)
Eosinophils (%)	0.00 (0.00-0.00)	0.00 (0.00-0.00)	0.00 (0.00-0.00)
Basophils (%)	0.50 (0.30-0.75)	0.40 (0.30-0.70)	0.60 (0.50-1.00)
Whole blood five days after admission			
White blood cells (10^3/uL)	9.34 (4.90-11.06)	7.92 (4.48-13.45)	9.48 (5.61-11.06)
Neutrophils (%)	6.75 (3.85-14.48)	13.65 (5.78-15.53)	4.40 (3.03-7.88)
Lymphocytes (%)	7.30 (4.45-10.50)	9.55 (7.10-13.35)	5.00 (3.38-7.38)
Monocytes (%)	1.10 (0.15-1.90)	1.10 (0.70-1.88)	0.65 (0.00-3.33)
Eosinophils (%)	0.25 (0.13-0.30)	0.25 (0.20-0.30)	0.20 (0.03-0.38)
Basophils (%)	0.12 (0.03-0.21)	0.15 (0.03-0.63)	0.12 (0.04-0.16)
PBMC five days after admission			
White blood cells (10^3/uL)	2.27 (2.01-3.39)	2.27 (2.10-3.35)	2.64 (1.03-3.39)
Neutrophils (%)	17.50 (8.58-22.48)	8.65 (7.60-34.53)	19.75 (16.50-22.48)
Lymphocytes (%)	52.95 (31.50-59.43)	47.85 (31.50-63.30)	57.65 (31.05-59.43)
Monocytes (%)	27.10 (23.60-44.08)	35.15 (26.00-44.08)	23.70 (20.13-46.03)
Eosinophils (%)	0.00 (0.00-0.00)	0.00 (0.00-0.00)	0.00 (0.00-0.00)
Basophils (%)	0.55 (0.12-1.28)	0.21 (0.01-0.48)	1.15 (0.68-1.48)

Supplementary Table 3: Genes displayed in heatmap of **Figure 2H.** Related to Figure 2.

Gene symbol	log2 fold change *	p-value
TREML4	-9.33709	2.14E-05
MIR7641-2	-2.47005	0.002589
LPCAT1	2.031801	3.97E-05
IL10	1.065163	0.001814
LINC00689	3.739978	0.002125
SLC51A	2.099609	0.004202
ADGRG3	1.673412	0.004231
ZDHHC19	2.823089	4.48E-06
MICB	2.585685	0.001359
LRG1	1.16365	0.000604
ANKDD1B	1.32709	0.002124
PIK3CD-AS1	0.88995	0.000178
IL1RN	0.809709	0.002971
IFIT1B	2.881311	0.003805
DDX60L	1.139357	0.001651
CBWD6	1.173252	0.000214
RBM43	0.970275	0.002312
WFDC21P	4.05669	0.000487
SYNPO2	1.670912	0.003177
SOBP	3.295889	0.001449
SUMO1P1	1.456744	0.000496
MGAT4A	0.789025	0.004923
KLRC2	2.1165	0.000793
AKAP5	2.440379	0.000192
CD72	1.394303	0.001677
PLAC8	1.464798	0.003344
SLC30A4	1.605307	0.004426
PNPT1	1.134624	0.004084
SH2D1B	1.823213	0.00027
IL18RAP	1.812693	0.000811
SLC35E2	-1.27329	3.08E-05
HM13	-0.48393	0.002582
GPR135	-1.3297	0.001644
LUC7L	-1.24367	0.001195
VDR	-1.57407	0.003439
TFAMP1	-4.7875	0.000228
TCEA2	-1.02761	0.003184
DNAJC7	-1.1878	0.002619
AVPI1	-0.90545	0.002113
PDXP	-0.84361	0.004749
CHEK1	-4.63781	0.000666
MBNL2	-1.80341	0.001966
CCDC86	-0.63525	0.003946

LOC100288152	-1.24735	0.000864
C1orf53	-1.97481	0.001537
ZNF668	-0.87961	0.000801
PANO1	-1.73433	0.002827
ANKDD1A	-1.03553	0.000492
ZBTB46	-1.06592	0.004639
RAD54L	-2.06287	0.003253
TONSL	-1.19694	0.002862
ROR2	-3.37008	0.002996
PIEZO2	-3.08025	0.002626
FZD7	-2.06404	0.00484
LOC100335030	-1.59151	0.000198
FANCA	-3.45732	0.001465
DNAH8	-2.72094	0.002291
CCNE1	-1.98496	0.001138
TK1	-1.7106	0.00409
HIST1H4A	-1.45902	0.000525
NOV	-3.80808	0.004211
SERPINB10	2.117877	0.001043
DDAH2	2.71082	0.004007
ADAMTS1	3.271828	1.06E-05
MS4A3	2.537861	0.001049
BPI	1.412597	0.001116
KLHL14	-3.15371	0.004334
CD27	-2.16616	0.003735
CCR10	-2.84861	0.001859
NUGGC	-2.09155	0.004026
HIST1H3G	-2.07989	0.003958
CDC20	-2.04678	0.003868
ZBTB32	-2.46777	0.002884
MYO1D	-2.4475	0.000606
GPRC5D	-3.17541	0.003406
TXNDC5	-2.50757	0.001479
RAPGEF5	-3.40663	0.00215
ANKRD36BP2	-2.24412	0.000637
GSG2	-2.04423	0.003735
HIST1H3B	-1.82588	0.002808
UCHL1	-2.96166	0.001272
PARM1	-2.0716	0.002834
CFAP54	-2.46088	0.003075
FER1L4	-4.27014	0.000583
IGLL5	-3.69963	0.000436
HID1	-3.06147	0.0003
TNFRSF13B	-2.98009	0.002759
GLDC	-3.29366	0.003695
JCHAIN	-2.18465	0.004266

LENG8	-3.51839	0.000504
SLC4A1	6.865651	0.002709
HBB	5.731114	3.29E-05
HBA2	7.043197	9.2E-06

* Log2 fold change: Difference between mean log2 fold change of gene expression in COVID-19 patients who recovered versus patients who required ICU admission or died.
Genes are listed in same order as shown in heatmap (**Figure 2H**) from top to bottom.

Supplementary Table 4: Genes displayed in heatmap of **Figure 3H.** Related to Figure 3.

Gene symbol	Log2 fold change	p-value
TREML4	-7.04571	6.61E-26
ADAMTS5	-3.74279	5.07E-07
CTTNBP2	-8.10413	3.91E-10
DUSP2	-2.68855	6.27E-05
CD1C	-2.17054	1.39E-06
ZNF703	-2.19917	0.000297
BFAR	-1.96088	0.000993
CCDC122	-2.04642	0.000635
HLA-DPB2	-2.39417	1.75E-06
ADGRE3	-2.90594	0.000351
HLA-DOA	-5.59538	0.000162
FAM167A	-3.67792	0.000476
HLA-DMA	-7.09137	1.99E-06
KNDC1	-2.74683	0.000952
RTN1	-1.86463	2.35E-06
ADGRD1	-1.53986	0.000826
HNMT	-1.31252	0.000553
TMEM86A	-1.23842	0.000431
OXGR1	-5.30736	2.54E-05
CACNA2D3	-1.90249	5.75E-06
CAMK1D	-2.19223	2.6E-13
C3	-2.19134	6.78E-09
CX3CR1	-1.77926	0.000325
HLA-DRA	-1.33564	0.000138
PAQR7	-1.15699	0.000558
ABCA9	-3.35174	0.000139
SLC35E2	-1.38076	2.77E-05
MB21D2	-1.42501	0.000519
SH3BP4	-1.75409	5.51E-07
KCNC3	-1.27091	0.000306
TSPYL5	-1.90701	0.000585
TBC1D9	-1.05018	0.000775
CD74	-1.02395	0.000459
ZBTB46	-1.48894	0.000428
ZNF395	-1.02917	0.00012
RPS3A	-0.84285	0.000972
LINC00936	-0.96803	0.000407
RAPH1	-1.40872	0.000812
IER2	-1.30069	0.000484
IL1R2	4.583973	8.03E-06
GLDN	4.462276	1.51E-05
IFITM1	2.736501	0.000328
ISG15	2.719541	0.000243

ANKRD45	5.380558	2.03E-06
NRIR	3.406254	6.13E-05
SIGLEC1	4.386509	0.000567
SLCO4A1	2.53741	3.47E-05
TIMP4	5.849213	0.00023
MFGE8	2.722993	0.000958
IL1R2	4.684606	0.000306
SRGAP1	2.70742	0.000141
ABTB2	2.189041	0.000859
NKD1	5.183029	0.000208
ANKRD22	2.060797	0.000151
TNFAIP6	1.670038	0.000728
TMEM45A	2.498901	0.000374
FMN1	1.769936	0.000023
ZNF608	1.484828	0.000615
LOC101927272	4.220622	0.00094
FRRS1	1.039967	5.66E-05
LINC01410	1.039229	9.71E-05
TMEM144	1.170582	4.04E-05
HIST1H2BD	1.422863	0.000344
P2RY1	1.057839	0.00018
LHFP	1.958464	0.000177
ADCY3	1.923302	2.33E-07
ASPH	1.658725	0.000786
THBS1	1.723182	0.000248
SOCS1	1.673436	0.000796
SSC4D	3.168443	6.62E-06
C3AR1	1.445453	0.000217
CREB3L1	7.571913	7.18E-07
TGM2	2.289047	0.000316
XK	2.412617	0.000266

* Log2 fold change: Difference between mean log2 fold change of gene expression in COVID-19 patients who recovered versus patients who required ICU admission or died.

Genes are listed in same order as shown in heatmap (**Figure 3H**) from top to bottom.
